# Human fMRI at 11.7T: Assessing feasibility, stability, and reliability on the Iseult scanner

**DOI:** 10.1162/IMAG.a.1362

**Published:** 2026-09-17

**Authors:** Joseph Obriot, Zaineb Amor, Matthias Serger, Rüdiger Stirnberg, Philipp Ehses, Malte Riedel, Tony Stöcker, Klass P. Prüssmann, Shajan Gunamony, Son Chu, Alexis Amadon, Alexandre Vignaud, Vincent Gras, Florent Meyniel, Franck Mauconduit, Caroline Le Ster, Nicolas Boulant

**Affiliations:** Université Paris-Saclay, CEA, CNRS, BAOBAB, Neurospin, Gif-sur-Yvette, France; Institut de neuromodulation, GHU Paris, psychiatrie et neurosciences, centre hospitalier Sainte-Anne, pôle hospitalo-universitaire 15, Université Paris Cité, Paris, France; Cognitive Neuroimaging Unit, INSERM, CEA, Université Paris-Saclay, NeuroSpin center, Gif-sur-Yvette, France; German Center for Neurodegenerative Diseases (DZNE), Bonn, Germany; Institute for Biomedical Engineering, ETH Zurich and University of Zurich, Zurich, Switzerland; Imaging Center for Excellence, University of Glasgow, Glasgow, United Kingdom

**Keywords:** ultra-high field MRI, fMRI, motion correction, parallel transmission

## Abstract

Ultra-high-field magnetic resonance imaging (MRI) promises major gains in blood oxygen level dependent (BOLD) sensitivity but introduces severe challenges such as RF field inhomogeneity, $B_{0}$ field inhomogeneity, motion and vibration-induced field variations. We report the first whole-brain resting-state and task-based fMRI experiments at 11.7T. A 3D-EPI sequence was optimized using tailored parallel-transmit RF pulses, gradient reshaping to suppress vibration-induced $B_{0}$ fluctuations, and prospective motion correction using servo navigation combined with retrospective phase equalization (PEERS). Four participants were scanned at 11.7T with whole-brain 1.2 mm isotropic resolution with servo navigation and PEERS that considerably reduced residual motion and increased temporal signal-to-noise ratio (SNR). The Default Mode Network in resting state could be detected and a physiological-noise-dominated regime was identified. Four additional but different participants were scanned at 7T for tSNR comparison and validation of the protocol, data quality, and processing pipeline. Task-based fMRI likewise was conducted at 11.7T and yielded robust, spatially specific activations across motor, visual, mathematical, and language networks, again with improved sensitivity and cleaner BOLD responses when using servo navigation and PEERS. These results demonstrate the feasibility and reliability of whole-brain human fMRI at 11.7T and constitute a first-quality control milestone to further increase resolution at that field strength.

## 1 Introduction

One of the main motivations for increasing the main magnetic field ($B_{0}$) in magnetic resonance imaging (MRI) is the supralinear gain in signal-to-noise ratio (SNR; Le Ster et al., 2022; Pohmann et al., 2016) and increased sensitivity to blood oxygen level dependent (BOLD) contrast (Duyn, 2012; van der Zwaag et al., 2009). Ultra-high field systems (UHF, ≥7T) allow to increase the spatial and/or temporal resolution by trading off the SNR. While 7T MRI systems are now commercially available and common in neuroscience research laboratories, the field strength was pushed to even higher levels with custom designs in the past two decades, reaching as high as 9.4T (Vaughan et al., 2006), 10.5T (He et al., 2020), and 11.7T (Boulant, Mauconduit, et al., 2024; Pittaluga et al., 2025). A new project to achieve 14T (Bates et al., 2023) is also on track to answer neuroscientific questions and move toward individualized analyses (Dumoulin & Knapen, 2023).

Such machines, however, remain scarce and many challenges arise for BOLD fMRI studies at these extreme fields. Tackling these challenges is an inherent part of leveraging those custom systems and great efforts have been made in that direction (Boulant et al., 2023; Qu et al., 2026). Among these issues, some are common to any imaging sequence at UHF, namely the inhomogeneity of transmission ($B_{1}^{+}$), sensitivity to motion, and the specific absorption rate limitation (SAR). Parallel transmission (pTx) has been developed to tackle the $B_{1}^{+}$ field inhomogeneity, allowing to design pulses that restore homogeneous excitation in an SAR-efficient way (Adriany et al., 2005; Gras et al., 2023; Padormo et al., 2016). Echo-Planar Imaging (EPI) sequences, widely used for fMRI, are susceptible to the main magnetic field inhomogeneities which are already an issue at 7T and become more severe at 11.7T.

In the context of fMRI, since BOLD variations are usually a few percent of the signal (van der Zwaag et al., 2009), it is of great importance to have a high temporal signal stability in the time series. This is usually estimated using the temporal SNR (tSNR, computed as the voxel-wise ratio between the mean and the standard deviation of the signal time series). Fluctuations in the signal are multi-factorial, incorporating both thermal and physiological noise, the latter being proportional to the signal magnitude (Krüger & Glover, 2001; Triantafyllou et al., 2016). But because tSNR does not always reflect the sensitivity of task signal response to BOLD contrast (Jamil et al., 2021; Le Ster et al., 2019), it is important to assess, in addition to tSNR, the detection of neural activations when benchmarking fMRI protocols.

While simultaneous multi-slice (SMS; Larkman et al., 2001) 2D-EPI has been the workhorse sequence to perform BOLD fMRI studies at high fields and up to 7T, 3D-EPI is, in principle, superior in SNR at high resolutions due to more noise averaging in the Fourier transform, and more SAR and acoustic noise efficient (L. Huber et al., 2018; Poser et al., 2010). It comes with the disadvantage of increased sensitivity to physiological noise in the data and to motion, both of which can reduce the tSNR when not corrected for Lutti et al. (2013).

Physiological noise includes motion and magnetic field changes induced by respiration, cardiac pulsation around vessels (in particular at the edges of the brain), spontaneous neural activity etc., and increases with magnetic field (Jorge et al., 2013; Triantafyllou et al., 2005). Some of these sources can be measured directly (e.g., breathing belt), or derived from the signal itself with assumptions of locality or periodicity (Behzadi et al., 2007; Glover et al., 2000). Prospective motion correction (PMC) and correction of field perturbations up to 1^st^ order can be achieved by servo navigation (Riedel, Ulrich, & Pruessmann, 2025; Serger et al., 2026; Ulrich et al., 2024), stabilizing the field of view (FOV) and shim settings during the acquisition. In a recent study, an additional retrospective phase correction method was proposed to further improve signal stability by tracking $B_{0}$ field variations across the time series and correcting for them (PEERS; Riedel, Ulrich, Bianchi et al., 2026). In this previous work Servo navigation and PEERS, however, were either benchmarked in anatomical acquisitions or in fMRI but limited to tSNR measurements.

First in-vivo experiments at 11.7T occurred in 2023 but were dedicated to anatomical acquisitions (Boulant, Mauconduit, et al., 2024). After results targeting this application were consolidated, a few resting-state fMRI experiments were attempted on the last 2 out of the 20 volunteers first authorized by the regulatory body, returning no identifiable default mode network and triggering a development and troubleshooting phase to recover the neural activations. Among these developments, the vibration-induced field fluctuations, that arise when mechanical resonances are excited, were targeted. As previously shown (Boulant et al. 2023), vibrations tend to increase linearly with field strength and can no longer be ignored at ultra-high field. Field oscillations not only can distort the k-space trajectory (L. Huber et al., 2025) but also can interfere with RF pulses and thus affect the flip angle and tSNR. Echo-spacings in EPI usually are tuned to avoid such mechanical resonances but an MR sequence can incorporate, besides the EPI train, other gradient events with broad frequency response overlapping sensitive frequency bands.

The aim of this study, therefore, was to assess and report the quality of whole-brain human resting-state and task-based fMRI data at 11.7T using a custom 3D-EPI sequence. This sequence was optimized in terms of vibrations and parallel transmission with tailored pulses employed to mitigate RF field inhomogeneity at 11.7T. Prospective motion correction and retrospective phase stabilization were evaluated. Additional tSNR and resting-state measurements were performed at 7T on different volunteers as a measure of the quality of the data and protocol.

## 2 Methods

### 2.1 Sequence

**Fig. 1.**
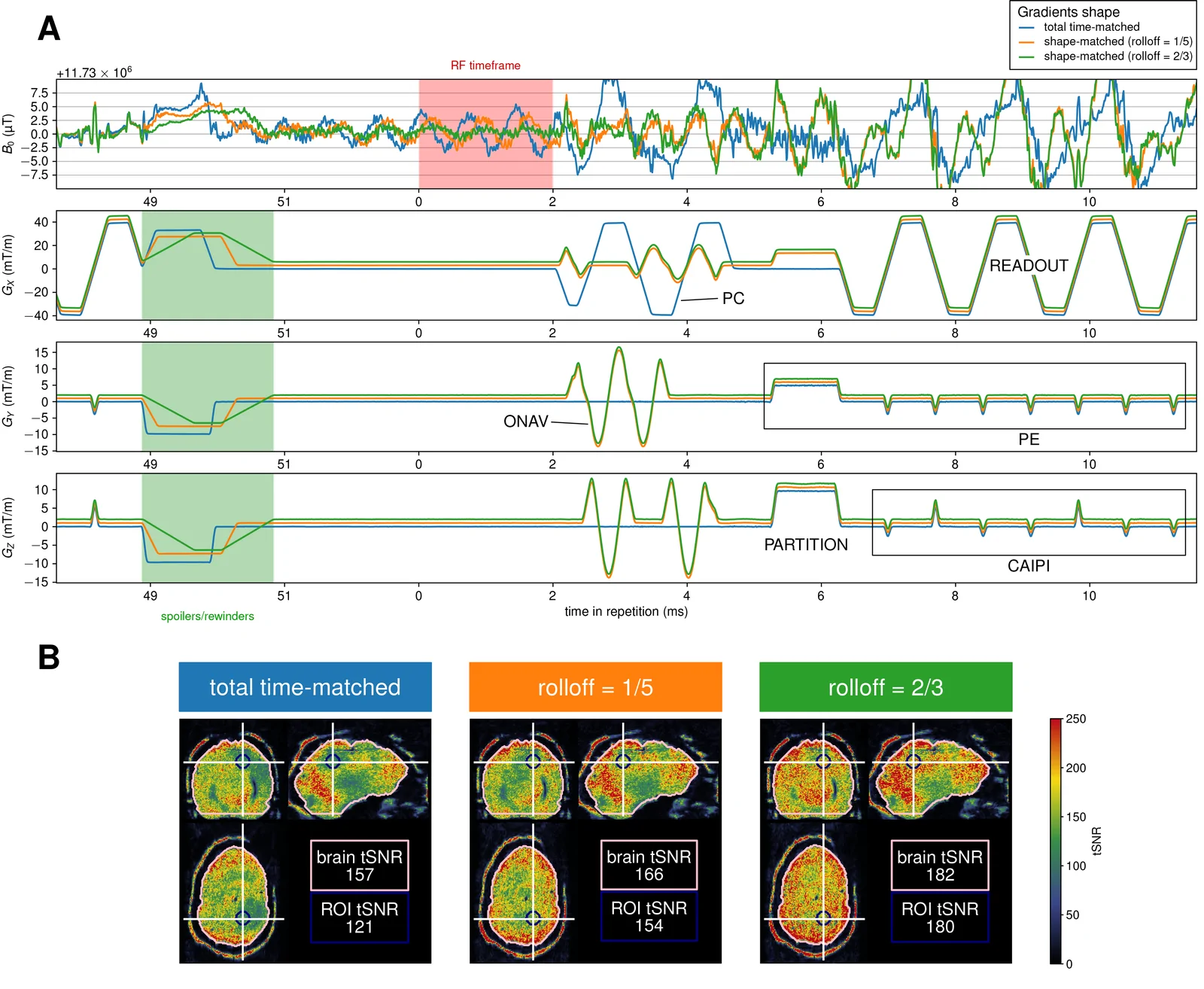
(A) Sequence events of the pTx and caipi-enabled segmented 3D-EPI sequence integrating servo navigation, recorded with field monitoring. The gradients measurement values are slightly offset vertically such that the otherwise overlapping curves are visible. Orbital navigators (ONAV) are visible and last approximately the same time as phase correction echoes (PC). Note that the last $G_{z}$ event in the TR shown is a rewinder, not a spoiler. No pulse was played for field recording. Initial $B_{0}$ measurements without gradient reshaping show fluctuations on the order of 8 µT peak-to-peak amplitude, after the spoilers and rewinders (blue), in the absence of gradient activity and within the 2 ms RF pulse time window. Careful design of the final gradient events of each TR through the rolloff factor leads to fewer oscillations. (B) This directly translates to tSNR maps showing higher values for lower $B_{0}$ oscillation amplitudes on a head-shaped phantom.

The CAIPI-enabled 3D-segmented EPI sequence (Stirnberg & Stöcker, 2021) used in this study is the one described in Serger et al. (2026), implemented on the VE12 Siemens platform. Notably, this sequence enables PMC and prospective correction of field perturbations up to first spatial order implemented through servo navigation (servoNav; Riedel, Ulrich, & Pruessmann, 2025; Ulrich et al., 2024), using ∼2 ms orbital navigators inserted every shot, immediately before the EPI readout train. When employing servoNav, the three echoes normally used for phase correction (PC; Chen & Wyrwicz, 2004) were only acquired once at the beginning of the time series instead of each shot to provide global N/2 ghost correction. In all subsequent shots, the three echoes were replaced by the orbital navigators, which were also used to update the frequency offset. The similar duration of PC scans and orbital navigators (Fig. 1A) allows for a consistent echo time (TE) whether using servo navigation or not in the sequence. A moving average filter (window size 812 ms) was applied to temporally smooth the servoNav field parameters in order to achieve more robustness.

### 2.2 Minimization of ${\text{B}}_{0}$ oscillations

At high field, rapid gradient switching during 3D-EPI sequences induces eddy currents and temporal fluctuations in the main magnetic field ($B_{0}$) due to vibrations (Boulant et al., 2023), including during the transition from the last gradient activity of the repetition time to the subsequent RF excitation (Fig. 1A). This can result in degradation of the excitation pattern, in particular when the RF pulse is long or when pTx pulses are used, because phase coherence is usually needed throughout composite shapes. To mitigate these effects that increase with the field and are thus particularly pronounced at 11.7T, the 3D-EPI sequence mentioned above was slightly adapted as follows:

- **Combination of spoilers and rewinders.**
  These gradient events were combined to leave time for $B_{0}$ oscillations to decrease until the next RF excitation.
- **Modification of the shape of gradient rewinders and spoilers.**
  These gradients (at the end of each echo train) could be modified at will in this custom sequence. The shape variations always maintained the same area under the curve so that zeroth order spoiling or rewinding moments were preserved. The gradient shapes could be optimized through the rolloff factor defined as ramp time/total time. Three scenarios were investigated:
  1. *Total time-matched*, corresponding to the implementation in the original sequence, which prepared all gradient objects such that they are as short in duration as possible but of same total time throughout the experiment (all phase and partition encoding steps) given 90% of the maximum slew rate and amplitude feasible with the utilised gradient system. The ramp times and durations differed on $G_{x}$, $G_{y}$ and $G_{z}$.
  2. *Shape-matched (rolloff = 1/5)*, corresponding to the optimized implementation, where not only the total time was matched, but the complete trapezoidal shapes were matched throughout aside from the amplitudes (ramp up = ramp down, ramp times are identical and flat top times are identical on $G_{x}$, $G_{y}$ and $G_{z}$). Therefore, also the hypothetical spectral responses would be matched, which—for a trapezoid—corresponds to a main sinc function, whose width is inversely proportional to the duration (ramp up + flat top time), multiplied by a second, wider sinc function, whose width is inversely proportional to the ramp time. These parameters can be used to tune the spectral response: via the duration, the first (and all other) zero crossings of the main sinc can be controlled; via the ramp time, an envelope function for the main sinc (and further zero crossings) can be controlled. The 1/5 rolloff gradient shape has a particular duration that places the first main sinc zero crossing at the dominant 1,350 Hz resonance peak on the z axis. But the ramp times are still relatively short, such that the first side lobe of the hypothetical spectral response is barely smaller than those of the main sinc without envelope (which would correspond to a hypothetical hard rectangular gradient pulse).
  3. *Shape-matched (rolloff = 2/3)*, again using the optimized implementation where the duration was maintained, but ramp time/duration = 2/3. This particular “rolloff factor” places the first zero crossing of the envelope sinc between the first and second zero crossing of the main sinc, etc. As a result, the hypothetical spectral response has minimized sidelobes.
- **Adapting spoiler.**
  While a gradient rewinder cannot be avoided, no spoiler was played on z as the $G_{z}$ → $B_{0}$ cross term (1^st^ → 0^th^ order spherical harmonic) contained a strong peak at the dominant vibration peak around 1,350 Hz, even with third order shim coils disconnected (Boulant, Le Ster, et al., 2024; L. Huber et al., 2025).

A dynamic field camera (Skope MRT, Zürich, Switzerland) was used to directly measure the temporal evolution of $B_{0}$ associated with the different gradient shaping schemes mentioned above. The resulting field recordings enabled assessment of field stability, guiding the selection of the gradient profiles that best suppressed residual field oscillations. The optimizations were performed on a head-shaped phantom, allowing characterization of $B_{0}$ fluctuations without participant-related motion or physiological noise. The impact of these optimizations on data quality was further evaluated by computing the tSNR in the phantom EPI time series.

The chosen *shape-matched (rolloff = 2/3)* gradient shape configuration provided the highest tSNR and the least $B_{0}$ fluctuations during the RF pulse time window (Fig. 1).

### 2.3 Retrospective phase correction with PEERS

The use of a moving average filter on the servoNav field parameters introduces a delay in field updates, which reduces the effectiveness of physiological noise correction. In addition, Figure 1 illustrates clearly how field fluctuations can differ between the navigator and imaging readout, reducing the effectiveness of the field correction. To counteract these effects and mitigate further physiological noise, retrospective phase equalization enhanced by run-time stabilization (Riedel, Ulrich, Bianchi et al., 2026) was additionally employed whenever servo navigation was used. This application takes advantage of the repeated, matching k-space lines enabled by prospective motion correction to derive zeroth and first order phase inconsistencies in k-space and correct them. This method was recently introduced and validated at 3T, but the evaluation of its performance was limited to tSNR comparisons.

### 2.4 Study design

The session consisted of two resting-state (rs-fMRI) series followed by two task-based BOLD fMRI runs, that is, one with servoNav (*Servo on*) and one without (*Servo off*) for each paradigm, with the order of the two conditions alternated and balanced between participants. During all resting-state acquisitions, participants were instructed to fixate a central cross to minimize eye movements.

The paradigm used for the task-based fMRI is a modified version of the functional localizer used in Pinel et al. (2007). It probes sensory, motor, and associative areas that are distributed over the entire brain. The modification of the original paradigm consisted of replacing the auditory stimuli with visual stimuli (the auditory sentences were displayed with rapid serial visual presentation), due to the lack of auditory stimulus setup. The localizer paradigm run was ∼5 minutes, comprising 100 events, including 20 rest periods. The remaining events comprised horizontal checkerboard stimuli (10), vertical checkerboard stimuli (10), mental calculation trials (20), language comprehension trials involving sentence reading (20), and motor trials corresponding to left (10) and right (10) button presses. The stimulus covered approximately a visual extent that corresponds to 0.02 steradians (around 9° horizontally and vertically).

### 2.5 Acquisitions

**Fig. 2.**
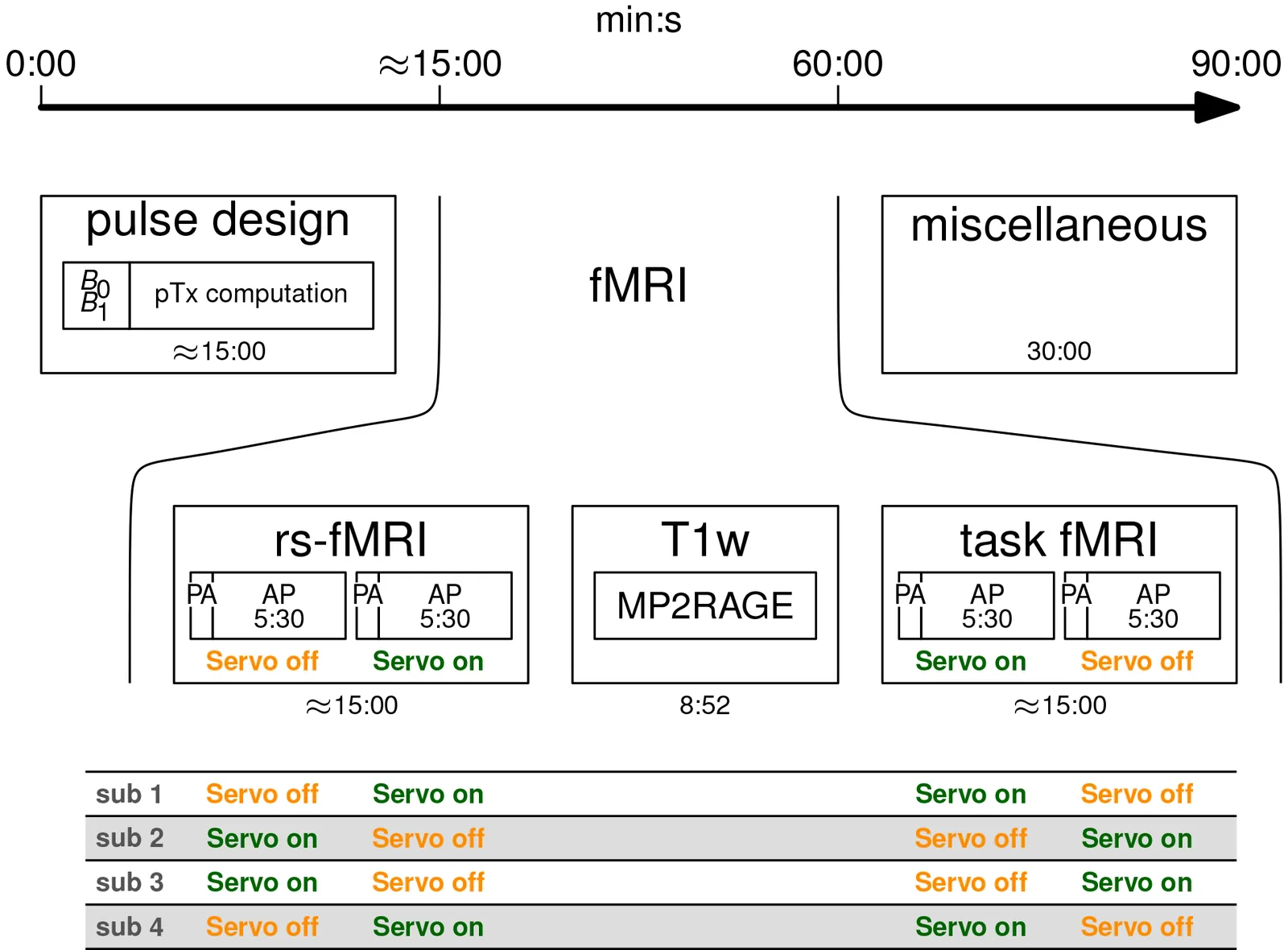
Session design. Timecourse of the imaging session including RF pulse design, anatomical acquisition, resting-state and task-based fMRI with alternating order of the scans acquired (with/without prospective motion correction, servoNav).

The study was conducted on the Iseult 11.7T scanner equipped with an 8Tx–31Rx RF coil (Chu et al., 2024). Measurements were obtained from four participants (right-handed males aged 19 to 24, hereafter referred to as participants 1 to 4). The protocol was approved by the French regulatory agency and an external ethics committee (Comité de protection des personnes, CPP, Sud-Est IV, reference n°100 074 TRISTAN), and written and informed consent was provided by all volunteers. Each participant completed a single scanning session conducted as described in Figure 2.

$B_{0}$ shimming was performed using a home made software that enabled shimming on a brain mask. The shim was performed up to the second order of the spherical harmonic decomposition (Boulant, Le Ster, et al., 2024). Resting-state and task-based fMRI scans were performed using the optimized 3D-EPI sequence described previously.

Imaging parameters were identical across rs-fMRI and task-based fMRI runs, with 155 volumes acquired over 5 minutes 30 seconds at 1.2 mm isotropic resolution, whole brain in a slightly tilted axial orientation with phase encoding in antero-posterior (AP). One volume with the phase encoding in PA was acquired just before the main series to estimate field-induced distortions. The FOV was 192 × 192 × 134.4 mm^3^ (112 slices, 7.1% slice oversampling). The shot TR was 53 ms (echo spacing = 0.71 ms), for a volume TR of 2.12 seconds (40 shots), a TE of 19.3 ms, and an FA of 13°, corresponding to the Ernst angle for gray matter at 11.7T ($T_{1}\approx$ 2.2 seconds). Parallel imaging was performed with a GRAPPA acceleration factor (AF) of 2 × 3 (in-plane × through-plane) using blipped-CAIPI sampling and 48 × 48 segmented EPI reference lines (Obriot et al., 2026), and a phase partial Fourier factor of 6/8. No fat saturation was applied, as it was found, still with field monitoring, that the fat saturation gradient spoiler likewise induced field oscillations and signal fluctuations. Minor adjustments were made with the EPI parameters for the fourth participant’s task run to avoid aliasing in the visual area due to the size of the head (less than 1 ms increase in TR_shot_ was applied to slightly increase the FOV).

To investigate the spatial distribution of tSNR, the spatial resolution was varied (1.2, 1.0 and 0.8 mm isotropic) on two participants using PMC. These acquisitions were designed to reduce single-voxel SNR and possibly observe different noise regimes. For this purpose, 53 volumes were acquired and the TR_volume_ was kept constant and TE as close as possible to the original one (1 mm acquisition TE = 19.3 ms, 0.8 mm acquisition TE = 21.8 ms) along with increased acceleration factors in order to match the desired resolution and TR_volume_ (1 mm acquisition AF = 3 × 3, 0.8 mm acquisition AF = 4 × 3). For this reason the 0.8 mm acquisition did not cover the whole brain in the z direction.

A $T_{1}$-weighted anatomical reference was acquired using the MP2RAGE sequence (Marques et al., 2010) along with retrospective fat navigators-based motion correction (Gallichan et al., 2016) at 0.8 mm isotropic resolution (TR = 5 seconds, TE = 2 ms, inversion times = 2.96 seconds & 964 ms, TA = 8 minutes 58 seconds, FA = 4° and 5°, AF = 4 × 1, 208 slices, FOV = 192 × 216 × 166 mm^3^).

For all servo-navigated fMRI acquisitions, the raw EPI time series data were first exported to apply the PEERS method offline (*Servo on + PEERS*), to correct for temporal field imperfections (drift or physiology-driven). The modified raw data were then re-imported to the scanner for vendor-provided image reconstruction (“IcePAT”).

### 2.6 Pulse design

For each participant a $B_{1}$ map was measured using the turbo-FLASH interferometric sequence at 2.5 mm isotropic resolution (TR = 20 seconds, TA = 4 minutes 30 seconds; Amadon et al., 2012). $B_{0}$ maps were also acquired at the beginning of the session using a three echoes GRE at 2.5 mm isotropic (TA = 35 seconds). They were used to calculate the pTx pulses as follows: for 3D-EPI a tailored slab-selective 2-ms pulse was designed for each participant using 4 k_T_-spokes of 500 µs each and time-bandwidth product equal to 15 (Cloos et al., 2012; Saïb et al., 2018), yielding an average flip angle (FA) normalized root mean square error (NRMSE) across participants’ brains of 14% (standard deviation 1%) and calculation time of 15 seconds. For the MP2RAGE, 7 k_T_ points were used for the small tip angle excitation (total pulse duration = 600 µs, computation time = 1 minute, NRMSE = 5.8%). The inversion pulse was designed using the fast gradient ascent pulse engineering method (fastGRAPE; Brégeat et al., 2024; Khaneja et al., 2005) approach with 19 basis coefficients for each gradient axis and each real/imaginary Tx channel waveform (total pulse duration = 4.1 ms, computation time = 5 minutes, NRMSE = 2.9%). Bloch simulation results of the flip angle for a representative participant are provided in Supplementary Figure S1 for the 3D-EPI pulses.

### 2.7 7T comparison

Additional measurements were performed at a different site (DZNE, Bonn, Germany) on a Siemens Magnetom 7T Plus scanner with a Nova 8Tx–32Rx RF coil (Nova Medical, Wilmington, MA, USA). Four different participants (hereafter referred to as participants 5 to 8) compared to the 11.7T scans were scanned at 7T. Here also, the protocol was accepted by the local ethics committee (Ethics Committee of the Faculty of Medicine, University Hospital Bonn, Study ID 191/13), and written and informed consent was provided by all volunteers. Each participant completed a single scanning session where only rs-fMRI data were acquired using the same 3D-EPI sequence and servoNav scheme as at 11.7T.

Acquisition parameters were identical to those used at 11.7T, except for a slightly longer echo time (TE = 22 ms) to match the Human Connectome Project protocol TE at 7T (Vu et al., 2017). As a consequence, shot TR was slightly increased (55.8 ms) and partitions marginally reduced (108 slices, 5.6% slice oversampling) to maintain almost the same volume TR (2.1204 seconds). Universal calibration-free pTx pulses were employed (Gras et al., 2017), with a mean FA-NRMSE of 11.5% on the 21 participants of the design database. Three hundred volumes were acquired to compare the speed of convergence of functional connectivity with 11.7T.

Here again, on two participants, three scans at increasing resolutions were performed for tSNR purposes, using parameters similar to the ones used at 11.7T except for TE. For 7T we chose a TE = 22 ms as for the resting-state acquisitions and, for further comparison, a TE = 28 ms as the $T_{2}^{*}$ of gray matter at that field strength (TE = 19.3 ms being the $T_{2}^{*}$ of gray matter at 11.7T used in our experiments; Norris & Ladd, 2023). A corresponding MPRAGE anatomical reference was acquired at 0.8 mm isotropic resolution again using universal pulses.

### 2.8 Preprocessing

For preprocessing, a standard procedure based on fMRIPrep was used (Esteban et al., 2019), with some adjustments to deal with the new 11.7T dataset.

- **Anatomical Images:** The MP2RAGE UNI images were denoised by multiplying the second inversion normalized magnitude image with the UNI image in a similar fashion to the MPRAGEise step of presurfer (Kashyap, 2021). Since the second inversion image retains the bias field (unlike the UNI image), and to avoid its reintroduction to the final image, ANTs’ N4BiasFieldCorrection (Tustison et al., 2010) was applied to it beforehand. Tests were performed to evaluate the quality of the segmentation of gray matter (GM) using fMRIPrep, FreeSurfer 7 (Dale et al., 1999; Reuter et al., 2012) and FastSurfer (Henschel et al., 2020), but all three produced unsatisfactory results, yielding massive segmentation faults in some participants. Better segmentations were achieved with FreeSurfer 8 which uses SynthSeg (Billot et al., 2023). We, therefore, ran the recon-all step of FreeSurfer (version 8) on each $T_{1}$-weighted image, outside of fMRIPrep, before passing these images to an fMRIPrep-based preprocessing pipeline, which included spatial normalization in the MNI152NLin2009cAsym template (Fonov et al., 2009).
- **Functional MRI:** A conventional fMRIPrep pipeline was used and included the following steps:
  - Rigid-body motion correction (Jenkinson et al., 2002) performed in a run-wise fashion with respect to a BOLD reference image with 6 DOF. This step was also performed for the *Servo on*, that is, motion-corrected, time series in order to evaluate and potentially correct residual motion.
  - Correction of susceptibility-induced geometric distortions using a $B_{0}$ fieldmap derived from pairs of AP/PA EPI images (TOPUP; Andersson et al., 2003). After rigidly aligning the estimated fieldmap to the aforementioned BOLD reference image, the resulting distortion coefficients were applied in the EPI (native) space.
  - Coregistration of the distortion-corrected BOLD reference image with the native $T_{1}$-weighted anatomical image using boundary-based registration (bbregister, FreeSurfer).
  - Computation of noise nuisance regressors, such as derivatives and quadratic terms of motion residuals, and physiological noise with CompCor decomposition (Behzadi et al., 2007) from the preprocessed BOLD time series.
  - Spatial transformations (head-motion transformation matrices, susceptibility distortion correction) and co-registrations to anatomical templates (native and MNI) were performed with a single interpolation step by concatenating all the transformations.

### 2.9 Analysis

- **Resting-state fMRI:** To retain only steady-state data, the first 5 volumes of the time series were removed. TSNR was calculated on the preprocessed data by clearing the signal of low-frequency fluctuations using cosine drifts equivalent to a high-pass filter with cutoff frequecy of 0.01 Hz. From the resulting data, the tSNR was calculated as the mean over the standard-deviation (μ/σ ) in MNI space.
  The default mode network (DMN) for every resting-state run was computed by applying the following steps:
  - A slight spatial smoothing using a 1.2 mm full width at half maximum (FWHM) Gaussian kernel was applied to the data for participant-level analysis.
  - The timecourse of a 4-mm radius seed in the posterior cingulate cortex (PCC) was extracted. The PCC was located either visually in the $T_{1}$-weighted space for results in this space or using known coordinates $\left(\;\begin{array}{c} 0\\ -53\\ 26 \end{array}\right)$ in the MNI standard space for results in the corresponding space.
  - The timecourse of this seed was then cleared of low-frequency fluctuations and nuisance regressors using up to 15 anatomical CompCor (computed from a principal component analysis on the white matter + CSF-masked data) and 25 motion regressors (translations, rotations, their derivative, quadratic and quadratic of derivative terms, and framewise displacement; Power et al., 2012) extracted by fMRIPrep. The choice of these regressors will be discussed further below. A design matrix was constructed using this seed timecourse and the same regressors, to be used in a general linear model (GLM). The effect size of the seed was computed and converted to z-statistic. Significance thresholds were set to |z|>3.9 (for false positive rate, FPR, less than 0.005% or p<0.00005 ) and |z|>3.29 (FPR at p<0.001 ) for individual participants and group respectively. Group maps were obtained by taking the average DMN z-scores across participants with comparable runs and at the same field, with the DMN computed directly in MNI space with smoother data (3 mm FWHM).

To assess the gain from the *Servo on + PEERS* implementation, the DMN coverage was estimated. It was computed by taking the number of voxels of the DMN maps in MNI space reaching the 3.29 z-score threshold within the DMN nodes of the Multi-subject Dictionary learning atlas (MSDL; Varoquaux et al., 2011) as the proportion of the total number of voxels in those nodes. For this metric, the data were smoothed again with a 3 mm FWHM Gaussian kernel to better match the spatial smoothness of such connectivity atlases and allow for group comparisons with matching coverage order of magnitudes.

The speed of convergence of the DMN extraction (the number of volumes needed to reach the same DMN coverage) was compared on the group level between 11.7T and 7T by using incremental number of volumes in the analysis. Given the small number of participants scanned at both fields, this was done as a quality control more than an inter-field comparison.

- **Task-based fMRI:** Voxel-wise tSNR maps were generated from the fMRIPrep preprocessed BOLD data after removal of task-related and drift components through regression (high-pass at 0.01 Hz). These participant-wise tSNR maps in the MNI space were averaged to produce the group tSNR maps.
  Functional MRI data were analyzed using a first-level GLM over the brain mask produced by fMRIPrep at the participant level. It included six task-related regressors, six motion regressors, cosine drift terms (high-pass at 0.01 Hz) and a baseline regressor. A first-order autoregressive noise model was used. For the *Servo on + PEERS* images, the motion regressors were derived from the servo navigation motion estimates. Since servo navigation provides prospective motion correction, the residual motion captured by fMRIPrep was minimal (less than 0.1 mm, cf. Supplementary Fig. S2). For scans acquired without servoNav, motion estimates derived from fMRIPrep were used. No smoothing was applied. The size effect maps were computed for 4 different contrasts: Click Right − Click Left (motor task), Vertical and Horizontal Checkerboards (visual task, cf. Supplementary Material), Calculations (mental calculations trials), and Phrases (language comprehension trials), the last two representing higher order cognitive tasks. After z-scoring the size effect maps, activation maps were obtained by thresholding the z-score maps for FPR at p<0.001 .
- **11.7T vs. 7T comparison:** 7T and 11.7T (respectively at two different sites) tSNR distributions were compared at different resolutions (1.2, 1, and 0.8 mm isotropic resolution, volume TR = 2.12 seconds) on four different participants (2 at each site).

### 2.10 Metrics

Resting-state and task-based (Localizer) fMRI images were used to evaluate the tSNR using both qualitative and quantitative measures. Temporal SNR was computed differently for resting-state and task-based data, as described in the previous subsection.

- Group tSNR maps in MNI space were generated in order to qualitatively compare the impact of *Servo on + PEERS* on tSNR against *Servo off*.
- Additionally, the impact of *Servo on + PEERS* on group and participant-wise tSNR distributions within the brain mask (in MNI space) was statistically quantified using paired t-tests. Temporal SNR distributions obtained from *Servo on + PEERS* data were compared against those obtained from the *Servo off* baseline data. The direction of the impact is indicated by arrows (↑ increase, ↓ decrease), and significance levels are indicated as * (p<0.05 ), ** (p<0.01 ), and *** (p<0.001 ).

The task-based fMRI data were used to assess the feasibility and reliability of 11.7T human fMRI. Likewise, the impact of *Servo on + PEERS* on sensitivity was evaluated using both qualitative and quantitative criteria:

- Thresholded activation maps in the native $T_{1}$-w space produced from the *Servo on + PEERS* data were visually compared with those obtained from the data acquired without servoNav for the four previously mentioned contrasts, namely, “Click Right vs Click Left”, “Checkerboard”, “Calculations”, and “Phrases”. The purpose was twofold: first, to confirm an adequate sensitivity for detecting robust activation patterns located in the expected regions for each of the contrasts, and second, to assess whether the application of servoNav and PEERS yielded higher sensitivity. The slices’ coordinates for the activation maps represent the same coordinates of the slices in MNI space. These coordinates were then projected back into each participant’s native space to make them comparable between participants, while remaining on the participant level in the native $T_{1}$-w space. They also represent relevant slices for each of the contrasts explored (motor, visual, mathematics and language-related regions).
- For each of the four contrasts explored in this work, and after thresholding the z-score maps for FPR at p<0.001 :
  1. The peak and median z-score values were extracted from the activation maps obtained from *Servo on + PEERS* and *Servo off* data sets for each participant and compared. Averages over the group were also computed for each metric and contrast. The peak z-score values were used as a simple descriptive statistic.
  2. Nonparametric permutation tests ($n_{\text{perm}}=10,000$ ) were performed over the median z-score values (*Servo on + PEERS* vs *Servo off* baseline). For the “Click Right vs. Click Left” contrast, z-score values related to the right click and to the left click were considered separately. The goal was to statistically quantify the increase or decrease in sensitivity associated with the *Servo on + PEERS* data relative to *Servo off* by assessing whether the observed differences in median values are statistically significant or could arise by chance. The results are indicated by arrows (↑ increase, ↓ decrease), and significance levels are indicated as * (p<0.05 ), ** (p<0.01 ), and *** (p<0.001 ).

Additionally, the impact of *Servo on + PEERS* on signal stability and noise levels was assessed by computing the mean percentage of BOLD signal change (%ΔBOLD ) within the regions of interest (ROIs) associated with the voxels corresponding to significant z-scores for the click right (z>3.29 , FPR at p<0.001 ) and click left (z<−3.29 , FPR at p<0.001 ) trials. After computing mean $\%\Delta {\text{BOLD}}_{\text{Right}}$ and $\%\Delta {\text{BOLD}}_{\text{Left}}$ , a summary plot was generated by plotting the difference between the two measures, that is, $\text{mean}\left(\%\Delta {\text{BOLD}}_{\text{Right}}\right)-\text{mean}\left(\%\Delta {\text{BOLD}}_{\text{Left}}\right)$. In the following, this metric is referred to as %ΔBOLD .

Finally, spatial specificity and consistency—particularly for the “Calculations” and “Phrases” activation patterns—were evaluated qualitatively by projecting the corresponding z-score maps onto each participant’s $T_{1}$-weighted native cortical surface and overlaying them with contours of the different parcels from the Destrieux Atlas projected into each participant’s native space (Destrieux et al., 2010). The Destrieux Atlas was chosen for its good spatial resolution and its relevance to both language and mathematics-associated regions. The threshold chosen for these surface-projected z-score maps was 2.5. Moreover, because the surface-based activation patterns for “Phrases” were relatively weak, an additional display is provided in Supplementary Material using a lower threshold of 1.5 and a different color bar for a better visualization of the potential spatial extent of the relevant activations. A key to understanding the Destrieux parcels’ mostly relevant to language and mathematics is also provided in Supplementary Material.

## 3 Results

**Fig. 3.**
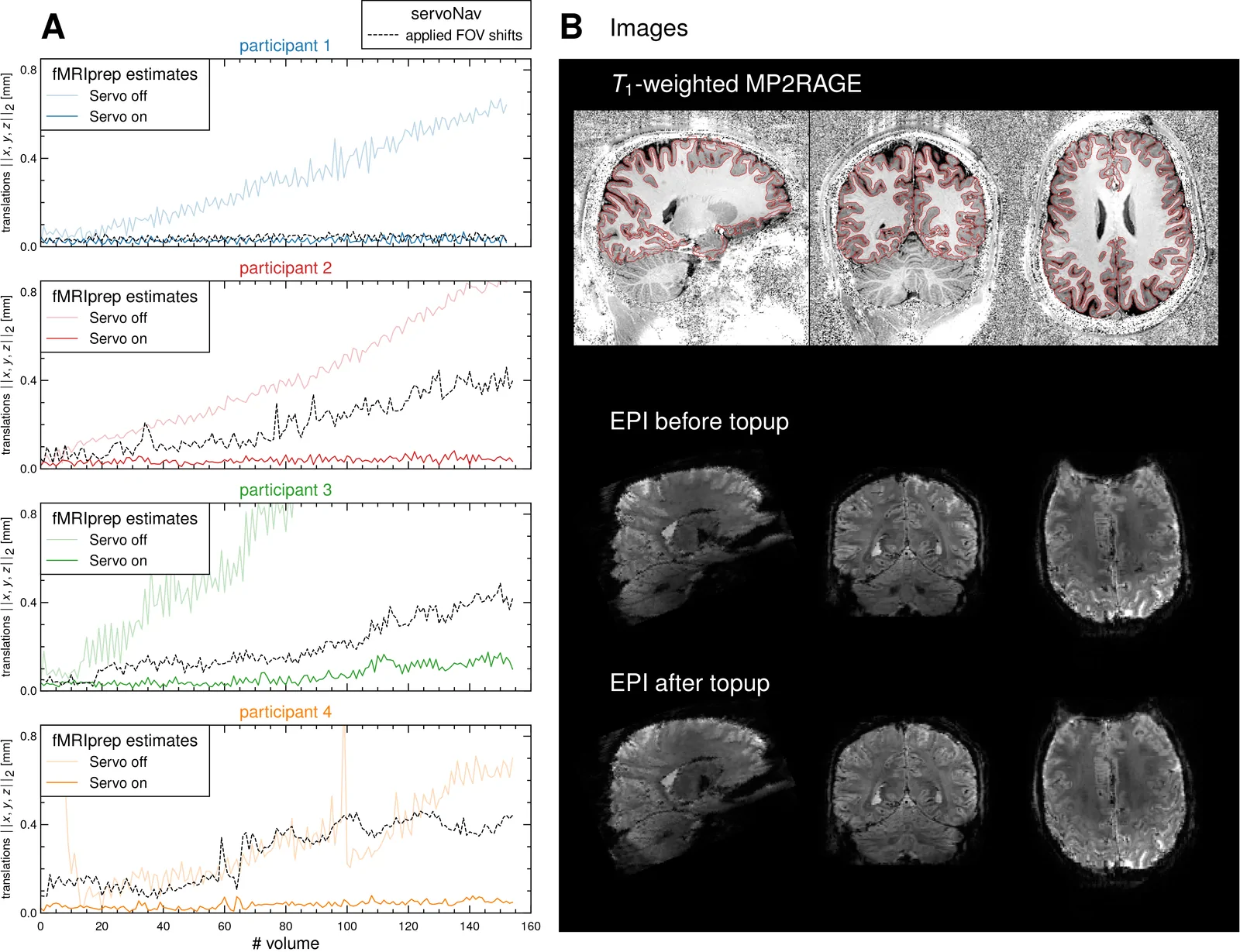
11.7T images quality and motion traces. (A) Translation estimates of the participants in resting-state runs, rotations have not been plotted to avoid cluttering the figure but are available in Supplementary Figure S3. The residual motion computed during preprocessing is shown in color for the *Servo on + PEERS* runs (vivid) and *Servo off* runs (faded). The FOV shifts applied in PMC are shown in dashed black lines. (B) Acquired images of a representative participant, $T_{1}$w slices are overlaid with cortical ribbon segmentation (red contour). EPI images are shown by pairs, before (top) and after (bottom) distortion correction. EPI images are from the servo-navigated runs.

Anatomical and EPI images acquired at 11.7 T are shown in Figure 3B. The images show minimal $B_{1}^{+}$-related artifacts typically expected at this field strength (e.g., hypersignal artifacts in MP2RAGE). The EPI images still suffer from expected geometric distortions in the AP direction (PE), especially in highly $B_{0}$ inhomogeneous areas such as above the sinuses in the prefrontal cortex. This is largely, but not completely, mitigated by TOPUP.

The realignment estimates of motion are also displayed in Figure 3A (obtained from the resting state data) and Supplementary Figure S2 (obtained from the task-based data), they show the *Servo on + PEERS* runs’ residual motion estimated by realignment are minimal, to the order of less than 0.1 mm (Table 1; Supplementary Fig. S2). Motion estimates from ServoNav are generally lower than retrospective estimates with *Servo off*, as the $\ell^{2}$-norm is partly driven by field drift–induced apparent y translations. The apparent motion is corrected by the frequency offset correction, and residual field fluctuations are further mitigated by PEERS during reconstruction. Supplementary Figure S3 and Table 1 show residual rotations are to the order of 0.1°. The reader should note that *Servo off* and *Servo on* correspond to distinct runs.

**Table 1.** Metrics returned by the analysis of the 11.7T resting-state runs.

	11.7T participants				
Metric	1	2	3	4	Group
**RMS displacement [mm]**	**0.03**	**0.04**	**0.08**	**0.04**	**0.05**
RMS displacement [mm]	0.36	0.48	0.93	0.41	0.55
**RMS rotation [°]**	**0.04**	**0.05**	**0.08**	**0.13**	**0.07**
RMS rotation [°]	0.05	0.26	0.68	0.15	0.28
**DMN coverage**	**45%**	**42%**	**53%**	**76%**	**56%**
DMN coverage	68%	66%	63%	61%	71%
**DMN coverage, no CompCor**	**57%**	**50%**	**56%**	**67%**	**60%**
DMN coverage, no CompCor	58%	55%	47%	60%	54%

RMS displacement here was calculated from fMRIPrep realignment estimates. Rows in bold correspond to *Servo on + PEERS* runs and regular rows to *Servo off* runs. No CompCor rows correspond to the same analysis as described in Section 2 except without using anatomical CompCor nuisance regressors, thus, in these rows, only movement nuisance regressors were used.

**Bold: *Servo on + PEERS***

Regular: *Servo off*.

**Fig. 4.**
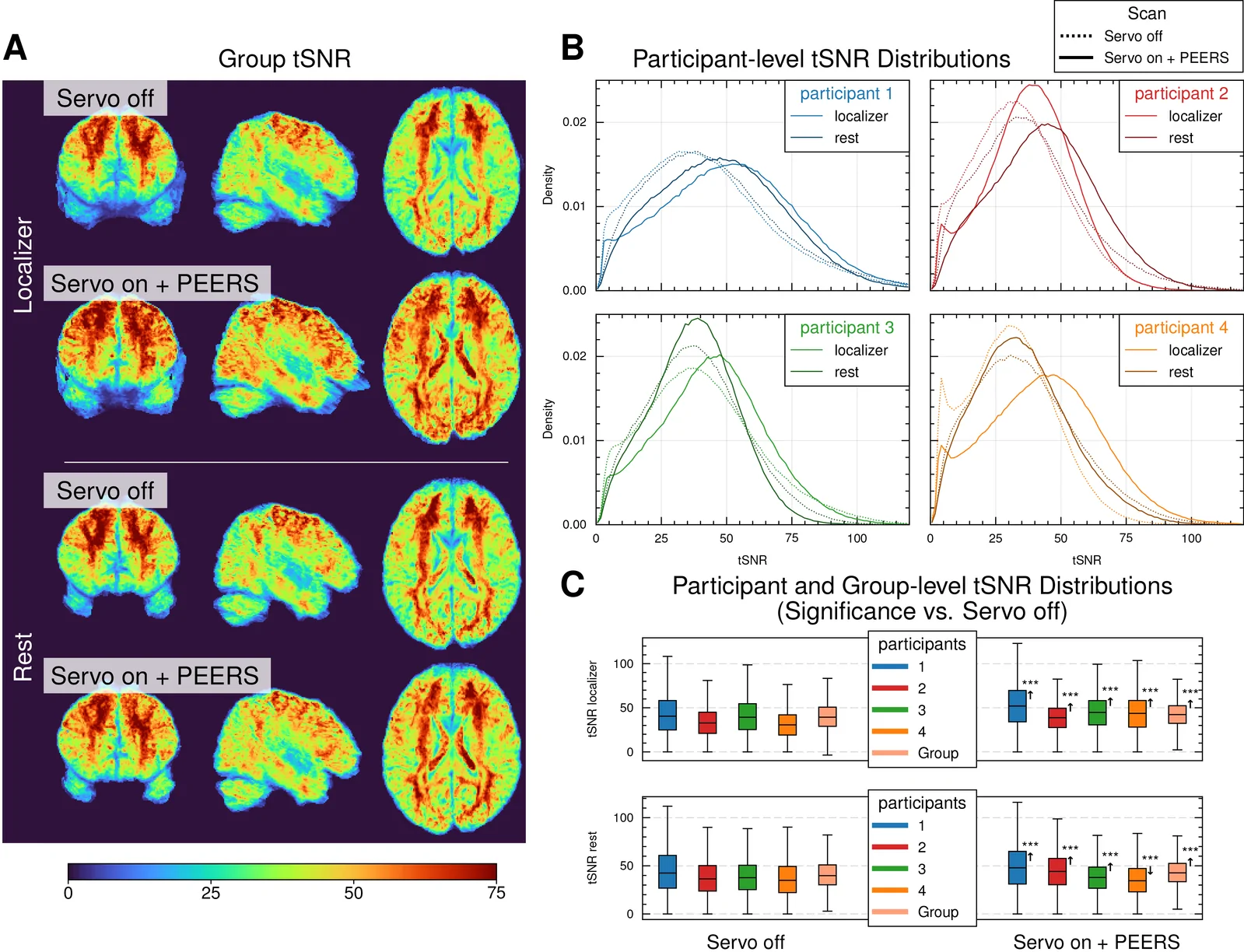
Temporal SNR. (A) Group tSNR maps of 11.7T participants in MNI space for the localizer and resting-state runs, with prospective motion correction using servoNav (*Servo on + PEERS*) or not (*Servo off*), after restrospective motion correction. (B) participant-wise distributions of tSNR, again for both localizer (vivid) and rest (dark) in the two conditions, *Servo on + PEERS* (solid) and *Servo off* (dotted). The corresponding participant-wise and group boxplots (C) reveal a significant increase in mean tSNR in nearly all comparisons (↑***) for the *Servo on + PEERS* condition versus *Servo off*. Boxes represent $Q_{1}$ until $Q_{3}$ and whiskers go up to the farthest point not exceeding $\left\{Q_{1},Q_{3}\right\}\pm 1.5\times \text{Interquartile distance}$ .

Group tSNR maps in MNI space, along with individual histograms of tSNR and comparison between *Servo on + PEERS* data and *Servo off* (after retrospective motion correction) data are presented in Figure 4A and B. Servo-navigated data always show a peak either equivalent or greater (shifted to the right) than *Servo off* runs, be it in resting-state or task-based (referred to as localizer) data. Additionally, Figure 4C shows that for all runs except the resting-state run for participant 4, a statistically significant increase in tSNR is associated with *Servo on + PEERS* relative to *Servo off*. On the group-level, this observation remains true as well.

**Fig. 5.**
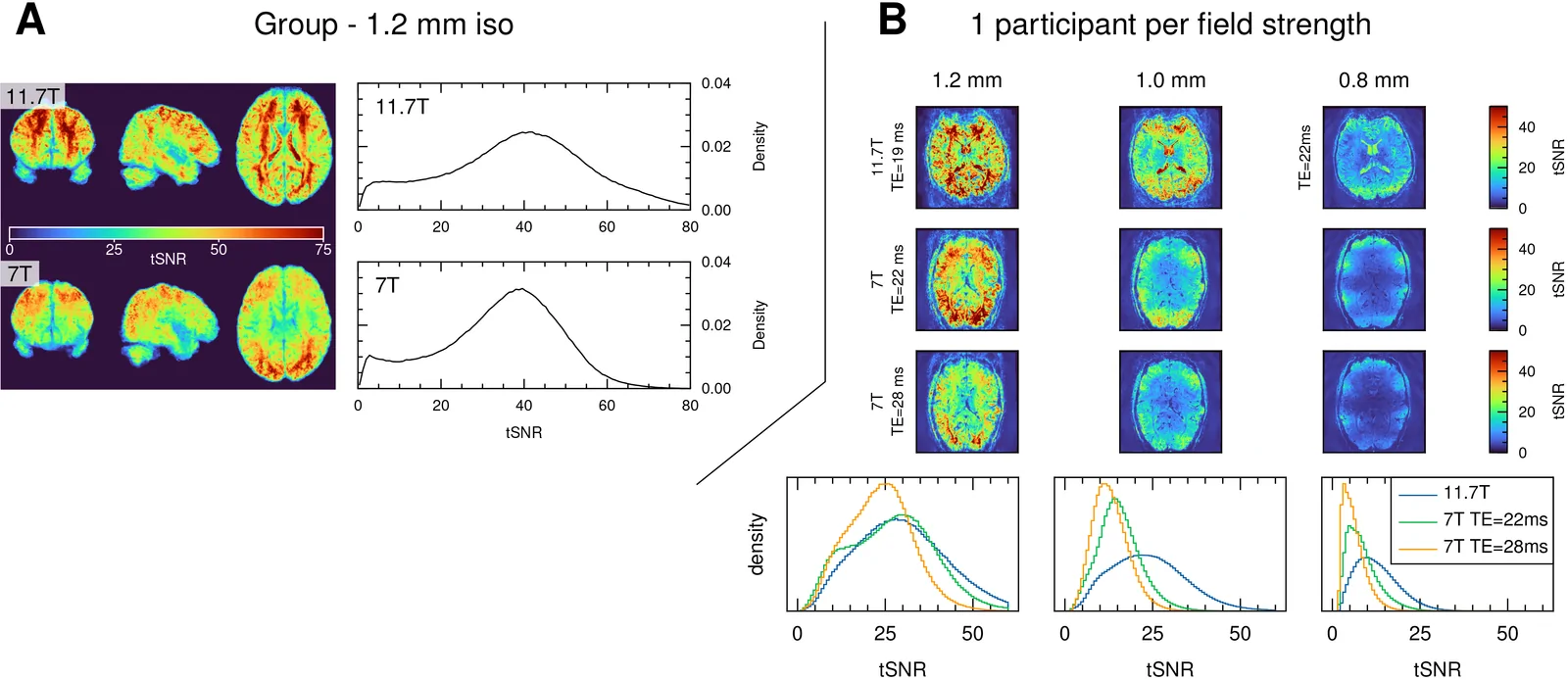
Temporal SNR at 11.7T and 7T. (A) Group tSNR maps calculated on resting-state runs employing servoNav and PEERS. Group tSNR distributions are revealing a similar peak value but a wider right tail at 11.7T. (B) A single participant per field strength scanned at increasing resolution revealing the crown-like pattern of tSNR—already present at 7T and 1.2 mm—appearing at a higher resolution at 11.7T (around 1 mm).

A comparison of tSNR at 1.2 mm between 7T and 11.7T data is presented in Figure 5A. While their peak values are similar, around 40, the spatial pattern of tSNR is very different between 11.7T and 7T, evidence that the noise regimes achieved at the two fields are not the same. Figure 5B shows a transition from physiological to thermal noise regime when going from 1.2 to 0.8 mm resolution at 11.7T on one participant, while at 7T the tSNR distribution patterns remain largely unchanged for the two TEs explored, only decreasing in intensity. At resolutions higher that 1.2 mm, the 11.7T participant exhibits a higher tSNR peak than the participant at 7T. 11.7T over 7T tSNR ratios reached respectively over 3 and 2 in the center and the periphery for the 0.8 mm acquisition and for TE = 22 ms at 7T. At TE = 28 ms, still at 7T, values reached respectively over 4 and 2.5. This observation is replicated on a second participant per field strength in Supplementary Figure S4.

**Fig. 6.**
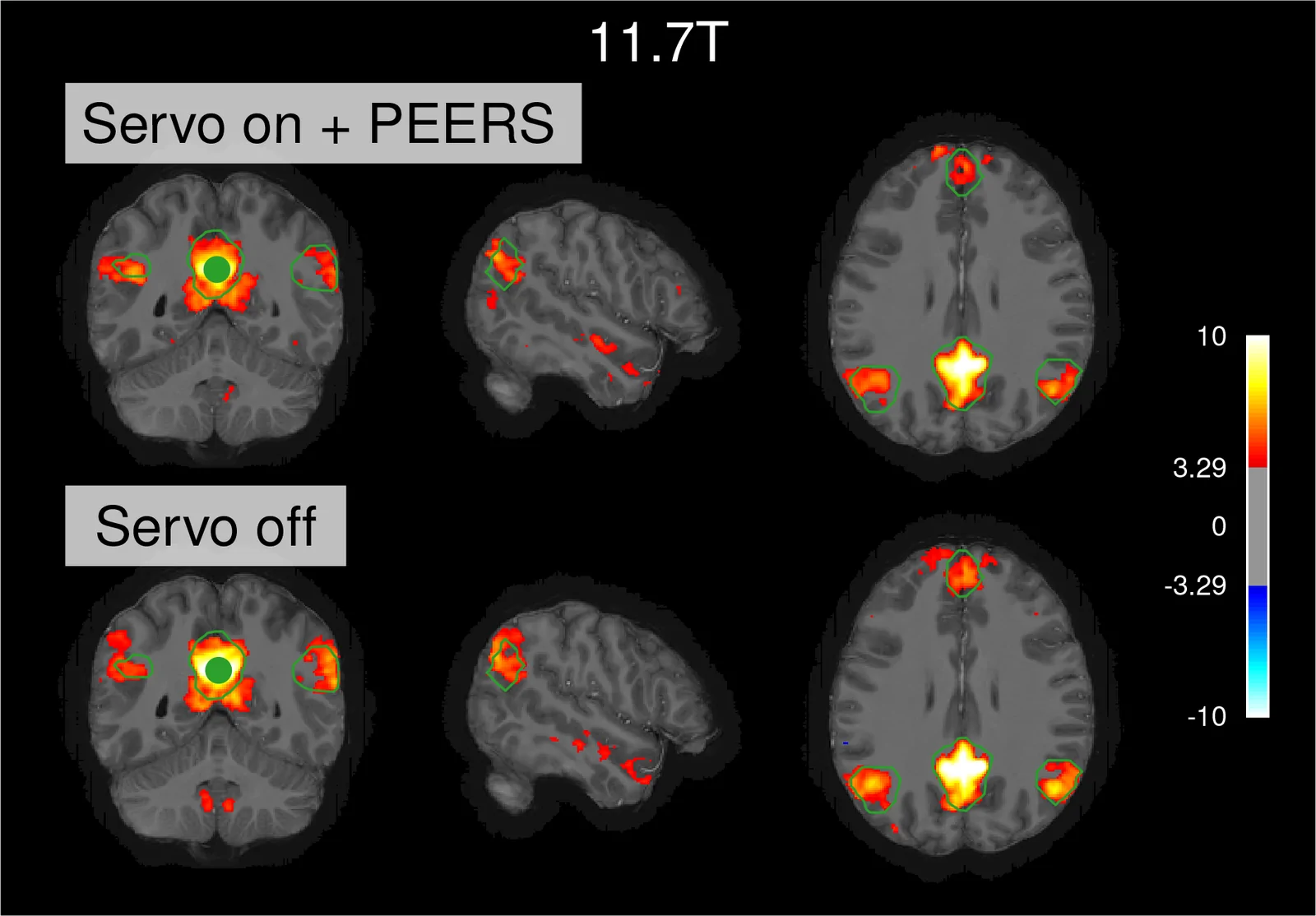
Effect of servoNav and PEERS on the default mode network at 11.7T. Group DMN maps at 11.7T again overlaid with the MSDL DMN nodes in green contours. The seed is visible on the coronal slices. This result was obtained after regressing out the CompCor nuisance components.

The comparison of the DMN between *Servo on + PEERS* data and *Servo off* is shown in Figure 6 and in Table 1. DMN patterns are hardly distinguishable while coverage is globally higher with *Servo off* when anatomical nuisance regressors are present. In the absence of such regressors *Servo on + PEERS* exhibits a greater coverage.

**Fig. 7.**
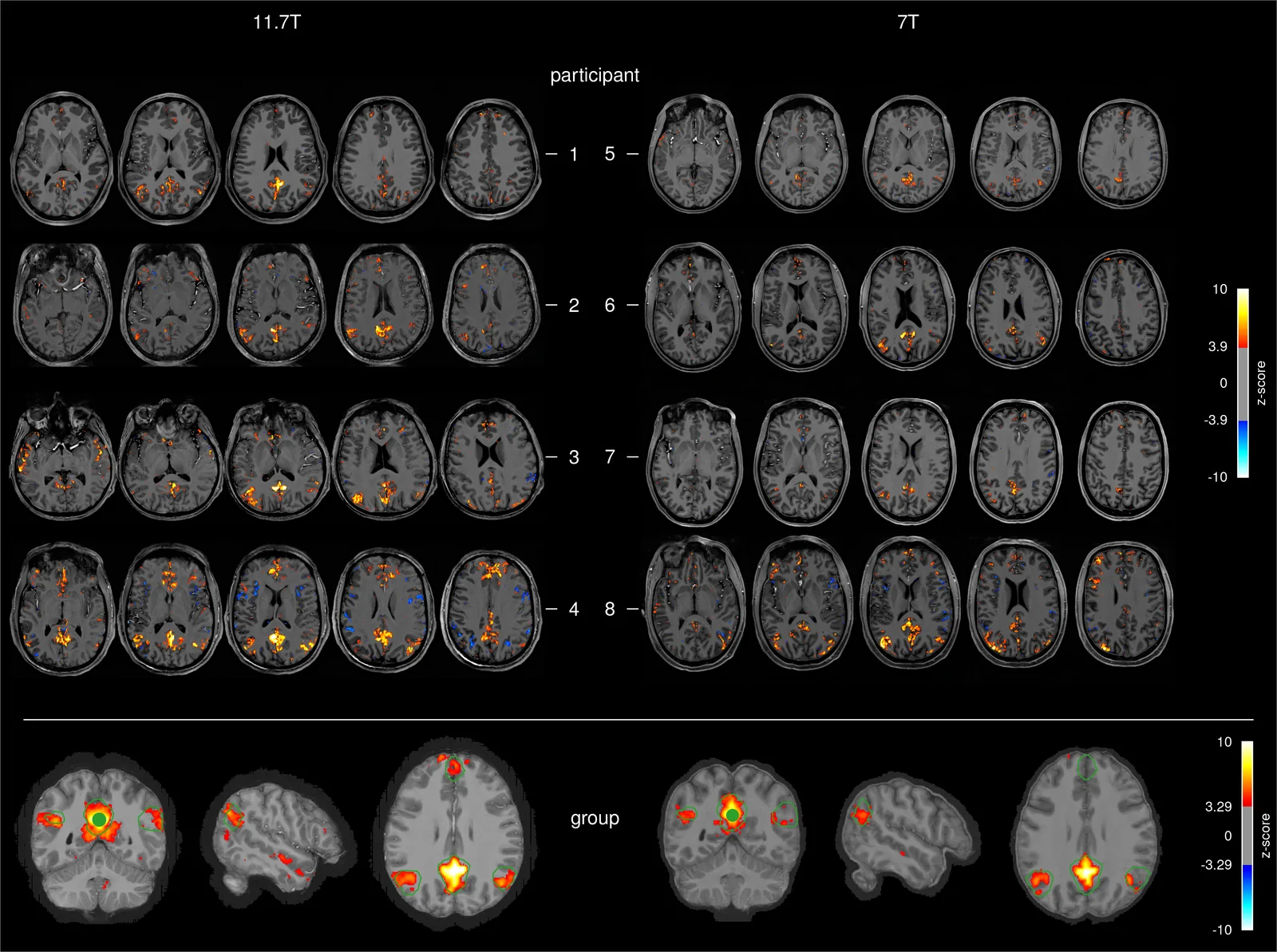
Default mode network at 11.7T and 7T, with servo navigation and PEERS. DMN maps in $T_{1}$ space for individual participants at 11.7T and 7T (participants are different between the fields), using the same number of volumes (150). The posterior cingulate cortex has been visually identified in $T_{1}$ space and a 4-mm radius sphere at its location was used as the DMN seed. The data was smoothed beforehand with a 1.2-mm FWHM kernel for the individual maps and a 3 mm one for the group maps. Green contours in group maps represent the DMN nodes of the MSDL atlas. The DMN seed (PCC) is visible in the group maps as a green dot in the coronal slice. Note the presence of activated voxels in the temporal pole at 11.7T, a known DMN area that is not present in the MSDL atlas.

Figure 7 shows individual and group level seed-based DMN maps, the main nodes of the DMN being identifiable in all participants at 11.7T while some expected nodes are absent from the 7T data, like the medial prefrontal cortex and temporal poles. The DMN coverage reaches up to 76% and 56% (Table 1) for individuals and group respectively (48% and 33% at 7T). The maps also show that the correlations are indeed specific to the DMN nodes and gray matter. Using more volumes recovers coverage at 7T (Supplementary Fig. S5). A group DMN coverage equivalent to 150 volumes (i.e., 5 minutes 18 seconds) at 11.7T is reached with around 250 volumes (i.e., 8 minutes 50 seconds) at 7T.

**Fig. 8.**
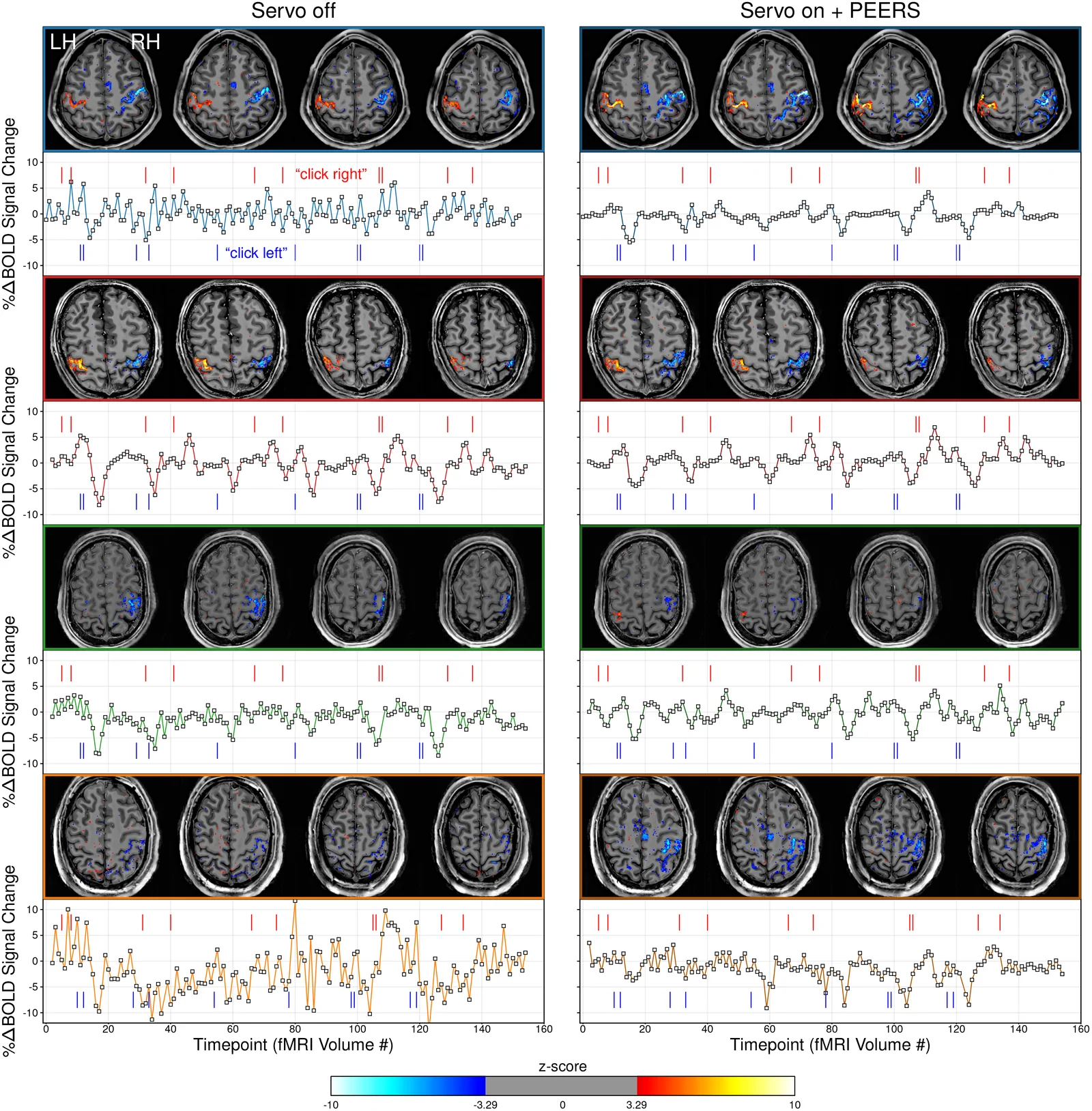
Participant-wise motor activation maps in the $T_{1}$-w native space and corresponding **%ΔBOLD **. Participant-wise statistically significant (FPR at p<0.001 ) z-score maps associated with the “Click Right vs Click Left” contrast and the corresponding %ΔBOLD . The slices shown represent the same slices in MNI space that were then projected back into each participant’s native space in order to facilitate the between-participant comparison in the native $T_{1}$-w space. %ΔBOLD is computed as $\text{mean}\left(\text{\%}\Delta {\text{BOLD}}_{\text{Right}}\right)-\text{mean}\left(\text{\%}\Delta {\text{BOLD}}_{\text{Left}}\right)$ within the ROIs associated with the significant z-scores for the click right and click left trials. From top to bottom: Participants 1–4.

Figure 8 shows well-localized motor activations that follow the motor cortex along the central sulcus especially in participants 1 and 2. Participants 3 and 4 have less clear activation patterns, especially for the right click: in right-handed participants, right click generally elicits less activation than left clicks. Supplementary Figure S6 shows well-localized visual activations in participants 1 and 2, less so in participants 3 and 4, for the “Checkerboard” contrast. This is mostly likely due to inter-participants variability: in fact, such inter-participant variability effect was also observed for the same “Checkerboard” contrast upon the reanalysis of one previously acquired dataset (Le Ster et al., 2019) at 7T with a nearly identical paradigm (the original Pinel et al. (2007) localizer without replacing the audio stimuli with visual ones).

The %ΔBOLD , corresponding to the difference between the mean BOLD signal change in the ROIs activated by right and left clicks, shows a signal peak (up to 5–7% of signal change) after each click delayed by the hemodynamic response: the activity elicited by most single clicks can be visually identified on the time series. The servoNav-associated plots exhibit less noisy signal variations that better fit the sequence of clicks. This is particularly noticeable for participants 1, 3, and 4.

**Fig. 9.**
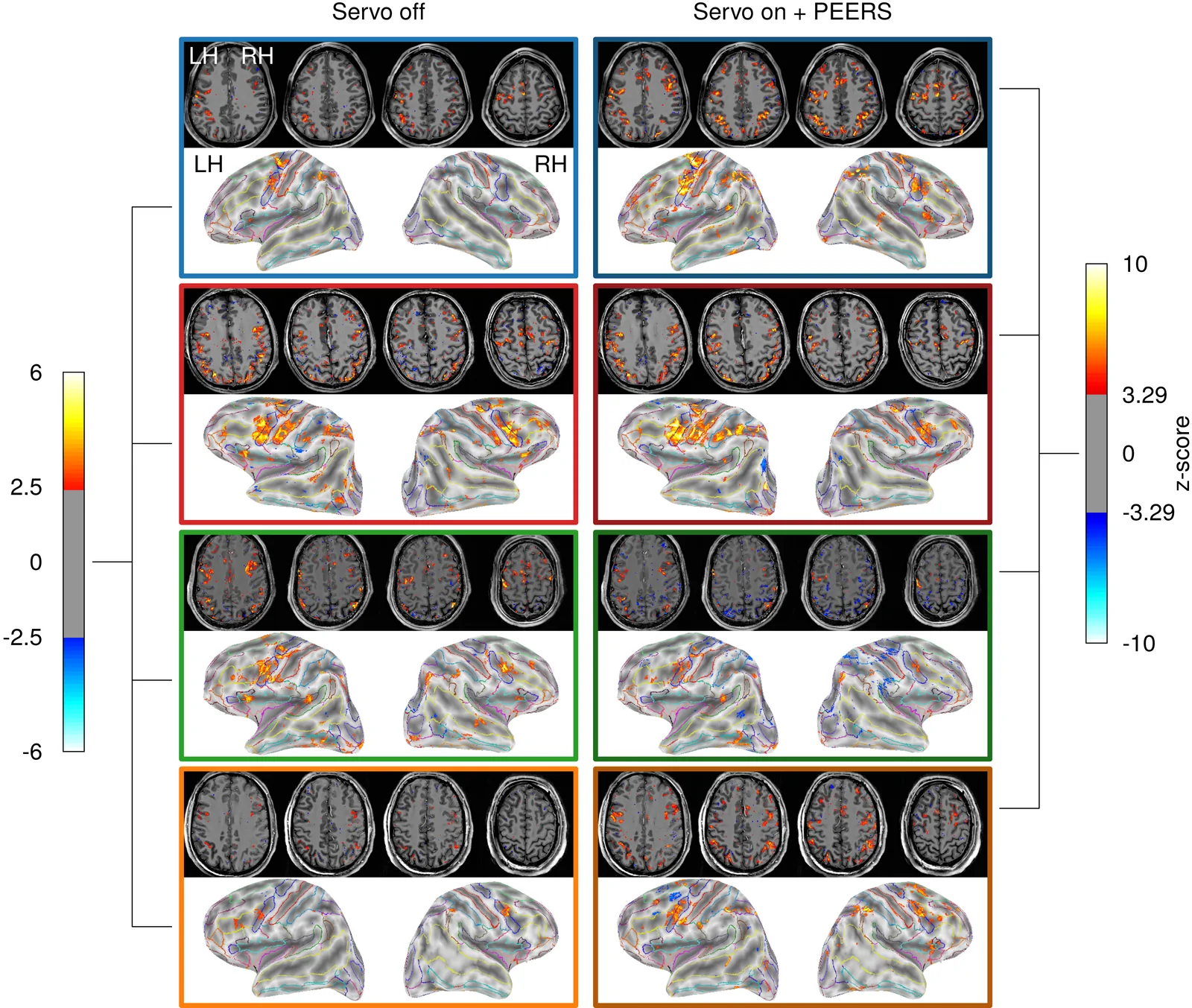
Participant-wise math-related activation maps in the $T_{1}$-w native space and their projection onto the native cortical surface. Participant-wise statistically significant (FPR at p<0.001 ) z-score maps associated with the “Calculations” contrast and their cortical surface projection. The contours of the parcels in Destrieux atlas projected into each participant’s native space are overlaid to the surface-projected activation patterns. The slices shown represent the same slices in MNI space that were then projected back into each participant’s native space in order to facilitate the between-participant comparison in the native $T_{1}$-w space. From top to bottom: Participants 1–4.

Volumetric and surface-projected activation maps associated with the math task—“Calculations”—(Fig. 9) reveal the expected cognitive areas for all participants at the single participant level. Most of the surface-projected activations patterns are located consistently at the intersection of precentral sulus and mid-frontal gyrus, and the intraparietal sulucs (IPS), cf. Supplementary Figure S7. Some language-related areas, such as Broca’s area and the posterior Sylvian fissure, are also activated in participants 1 and 3. This can be explained by the fact that the participants must read the subtraction instructions on the screen to perform it. Notably, the regions activated across participants are highly consistent: globally they overlay with the same parcels from the Destrieux Atlas. Visually, *Servo on + PEERS* seems to improve the results for all participants except the third.

**Fig. 10.**
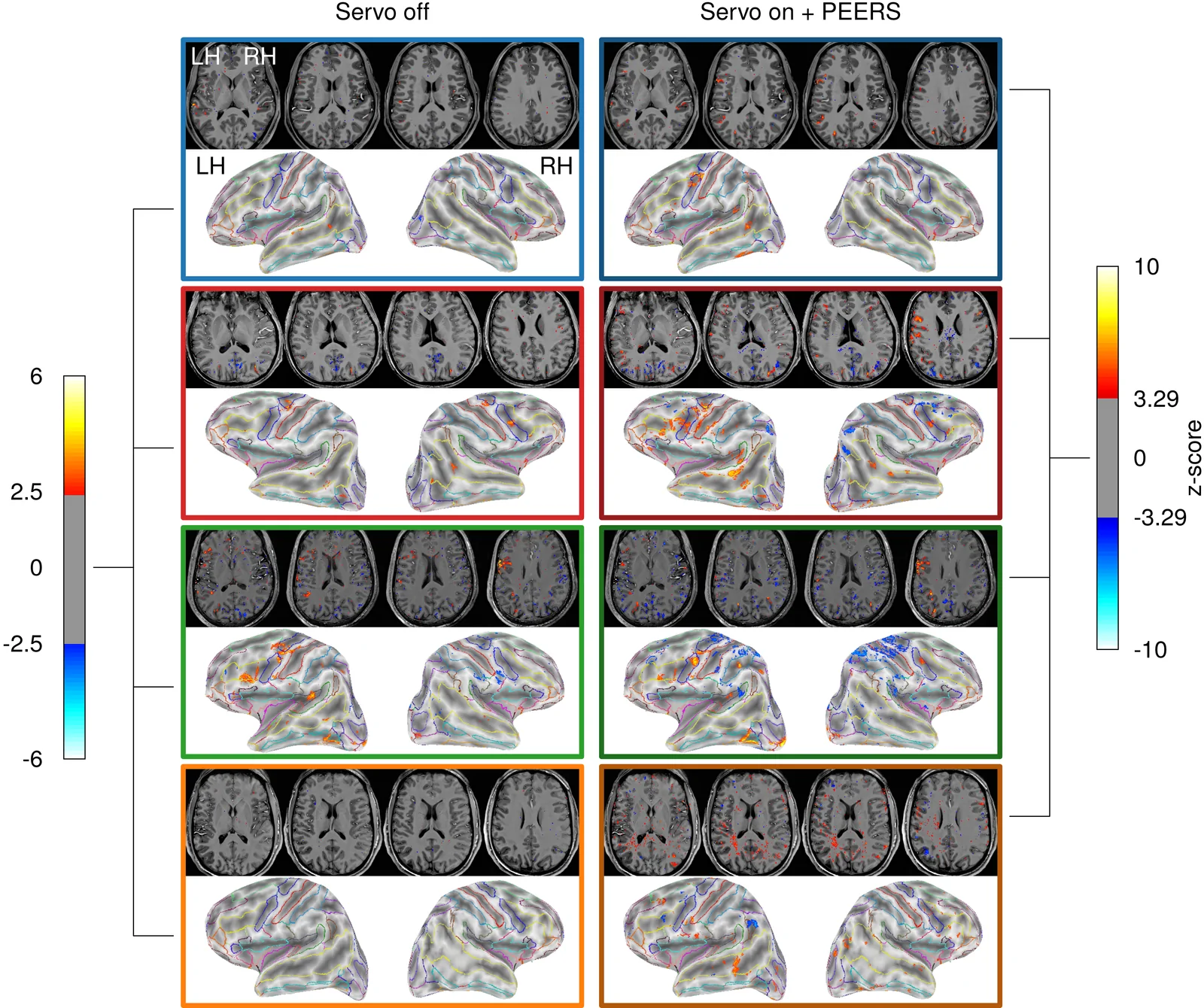
Participant-wise language-related activation maps in the $T_{1}$-w native space and their projection onto the native cortical surface. Participant-wise statistically significant (FPR at p<0.001 ) z-score maps associated with the “Phrases” contrast and their cortical surface projection. The contours of the parcels in Destrieux atlas projected into each participant’s native space are overlaid to the surface-projected activation patterns. The slices shown represent the same slices in MNI space that were then projected back into each participant’s native space in order to facilitate the between participant comparison in the native $T_{1}$-w space. From top to bottom: Participants 1–4.

For the participants 1, 2, and 3, the volume-based activation patterns associated with the language comprehension tasks are located in known language-associated locations (Fig. 10). However, the results are less significant than those associated with the math task. When projected onto the cortical surface, these volumetric maps (language-task related) appear even less significant, especially when servoNav and PEERS were not applied. Supplementary Figure S8 obtained with a lower threshold shows activations within the superior precentral sulcus, the superior temporal sulcus, the posterior Sylvian fissure, and around Broca’s area (cf. Supplementary Fig. S7). Of note, the activations were mostly left lateralized, consistent with the language network. Additional activations are also present in occipital regions, likely reflecting visual processing, as participants needed to see the phrases in order to read them.

**Fig. 11.**
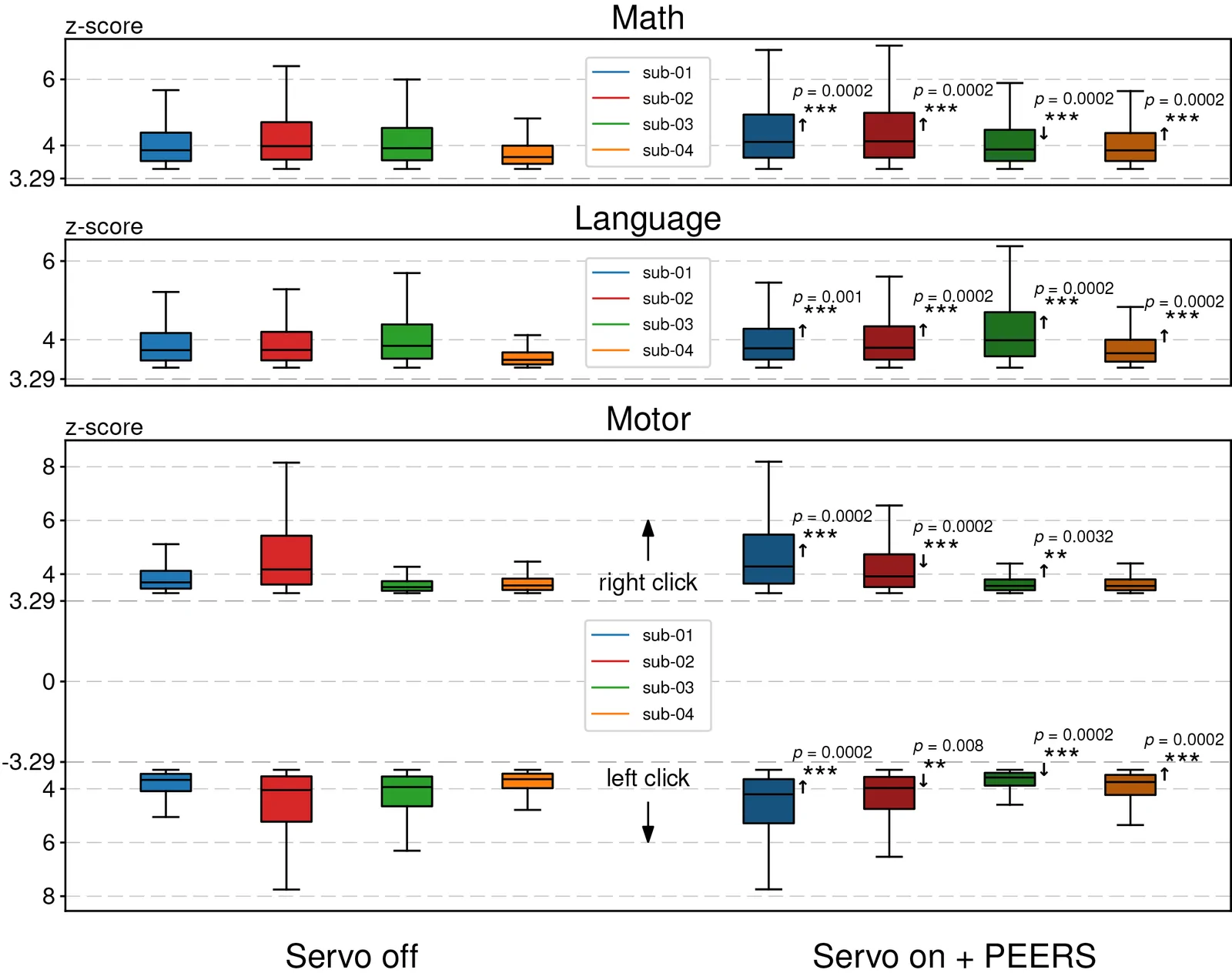
Participant-wise boxplots of the significant z-scores distributions associated with the “Calculations”, “Phrases”, and “Click Right vs Click Left” contrasts. The median of statistically significant z-score (FPR at p<0.001 ) values obtained from *Servo on + PEERS* data were compared to those obtained from the *Servo off* baseline data using permutation tests. For the “Click Right vs Click Left” contrast, significant z-score values related to the right click and to the left click were considered separately.

To evaluate a change in sensitivity between *Servo off* and *Servo on + PEERS*, a descriptive summary of the participant-wise and group-averaged peak and median z-score values for the different contrasts considered in this work is proposed (Table 2). On average, *Servo on + PEERS* always yields higher z-scores values. On the participant level, with the exception of the ‘Click right vs. Click left’ contrast, for which the results are mitigated, *Servo on + PEERS* runs yield higher values at least for participants 1, 2 and 4. These findings are consistent with visual inspection of the activation maps (Figs. 8, 9, and 10; Supplementary Fig. S6). Second, we assessed whether the observed differences in median (Fig. 11) values between *Servo off* and *Servo on + PEERS* are statistically significant using permutation tests. This metric suggests an increased sensitivity in most cases when using servoNav and PEERS for motor, checkerboard, and association areas (calculation, reading). For the “Calculations”,“Phrases” and “Checkerboard” contrasts, the application of servoNav and PEERS improves BOLD sensitivity for almost all participants, irrespective of the run order. For “Click Right vs Click Left”, the impact of servoNav and PEERS is mitigated. These results are in agreement with Table 2.

**Table 2.** Summary table of participant-wise and group-averaged peak and median z-score values, reported separately for *Servo on + PEERS* and *Servo off* and each of the 4 contrasts explored namely, “Click right vs Click left”, “Checkeboard”, “Calculations”, and “Phrases”.

		*Servo off*					*Servo on + PEERS*				
		Participant					Participant				
Contrasts	Metrics	1	2	3	4	Average	1	2	3	4	Average
Click right	peak z-score	8	**9.7**	**5.6**	5.9	7.3	**10.8**	8.1	5.5	**6.4**	**7.7**
	median z-score	3.7	**4.2**	3.5	**3.6**	3.8	**4.3**	3.9	**3.7**	**3.6**	**3.9**
Click left	peak z-score	-9.1	**-11**	**-8.3**	-5.9	-8.6	**-12.7**	-8.3	-6.5	**-9.3**	**-9.2**
	median z-score	-3.7	**-4**	**-3.9**	-3.6	-3.8	**-4.2**	**-4**	-3.6	**-3.7**	**-3.9**
Checkerboard	peak z-score	9.6	9.2	6.4	5.2	7.6	**9.8**	**9.4**	**6.9**	**6.9**	**8.3**
	median z-score	3.7	4	**3.6**	3.5	3.7	**3.9**	**4.2**	**3.6**	**3.7**	**3.9**
Calculations	peak z-score	8.2	9.9	**10.4**	6.2	8.7	**9.2**	**10.6**	8.5	**7.8**	**9**
	median z-score	3.9	4	**3.9**	3.6	3.9	**4.1**	**4.1**	**3.9**	**3.9**	**4**
Phrases	peak z-score	7.4	7.5	**10.2**	4.8	7.5	**7.6**	**8.2**	8.9	**7**	**7.9**
	median z-score	3.7	3.7	3.8	3.5	3.7	**3.8**	**3.8**	**4**	**3.7**	**3.8**

The higher value between *Servo on + PEERS* and *Servo off* runs for each participant and group-averaged is highlighted in bold.

## 4 Discussion

This paper presents the first human fMRI experiments at 11.7T, with an assessment of PMC based on servo navigation and retrospective phase equalization with PEERS for BOLD detection and tSNR.

Homogeneous RF excitation was achieved in the whole brain, including the cerebellum. Due to the small flip angles used in the 3D-EPI sequence, the SAR was relatively low. A next step includes designing universal pulses to make them accessible to standard users of the platform and gain acquisition time (∼15 minutes).

In a recent study, servo navigation was shown to improve image quality in high-resolution $T_{2}^{*}$ weighted EPI at 7T and 11.7T, provided adequate filtering of the servoNav parameters is applied (Serger et al., 2026). The results of this work indicate that the same setup is suitable for fMRI studies. However, the sequence’s sensitivity to field fluctuations required conservative temporal filtering of field updates, introducing a delay. PEERS can retrospectively mitigate the resulting phase inconsistencies of the frequency correction (including the lag), underscoring its importance for stabilizing the 3D-EPI time series measurements. This approach reduces the sensitivity of 3D-EPI to physiological noise, and comes at little cost as the navigators can be inserted in place of phase correction echoes. This approach requires no external device while estimating parameters with a high temporal resolution. Interestingly, in our case, Servo-navigation with PEERS almost systematically returned higher tSNR compared to no PMC. This is contrasted with results reported by Zaitsev et al. (2017) showing for 7 participants out of 11 deterioration of mean tSNR with PMC based on optical tracking. Although the exact reasons remain unknown, our data could suggest that further post-processing aiming at stabilizing the data, such as PEERS, is necessary to achieve more robustness. Since tSNR is an incomplete fMRI data quality metric, it appeared important to us to benchmark these methods with actual fMRI paradigms and activation maps.

For fMRI preprocessing, we kept the pipeline as standard as possible. Despite strong distortions that were not fully corrected with TOPUP, registration to the anatomical image was satisfactory across all participants. To avoid heavy interpolation during distortion correction, one may consider not correcting at all, using a distortion-matched structural image as anatomical reference instead (Kan et al., 2025). Other approaches to improve data quality could be explored, such as dual-polarity GRAPPA (Hoge & Polimeni, 2016) or FRISGO (R. Huber et al., 2025). Even with the servoNav, small residual motion persisted in the volume time series according to the fMRIPrep results, in particular in high motion scans. Retrospective motion correction was thus necessary and applied during preprocessing.

The tSNR patterns at 11.7T and 1.2 mm resolution revealed values in the white matter greater than in gray matter. This is reminiscent of a physiological noise dominated regime in the gray matter (Wald & Polimeni, 2017) at the 1.2 mm resolution of this work. This moderately high resolution was chosen on purpose because of the challenges encountered in initial tests (RF field inhomogeneity, motion, vibrations) to establish the feasibility of fMRI at 11.7T without adding extra difficulties in the post-processing and analysis. By extrapolating tSNR measurements at 7T at 1.6 mm resolution in Le Ster et al. (2019) and Gras et al. (2019), and assuming a nearly linear SNR gain in the brain periphery at 11.7T versus 7T (Guérin et al., 2017), such a noise regime at 11.7T at 1.2 mm resolution was not anticipated. We can tentatively explain this behavior 1) with recent SNR measurements showing superior gains than expected throughout the brain (factor of 3 in the center, >2 in the periphery; Chu et al., 2026), confirmed by the tSNR measurements at 0.8 mm resolution (Fig. 4; Supplementary Fig. S4) increased BOLD variance with field strength (Triantafyllou et al., 2005; Triantafyllou et al., 2016). At 7T the tSNR patterns clearly look different, with gray matter bearing higher tSNR levels, suggesting the predominance of thermal noise over physiological noise. This indicates that a thermal noise dominated regime (which is desirable) could be reached at a lower ${\text{SNR}}_{0}$ at 11.7T (Triantafyllou et al., 2005). An easy way to achieve lower ${\text{SNR}}_{0}$ while keeping gray matter tSNR in the periphery is to reduce the voxel volume up to the thermal noise domination threshold, thereby showing great promises for pushing the resolution of fMRI scans at 11.7T. The thermal noise threshold appears at a higher resolution compared to 7T, as evidenced by the tSNR patterns showing a change in pattern with increasing resolution at 11.7T while the patterns at 7T remained similar, suggesting for 11.7T a thermal/physiological regime frontier occurring at around 1 mm isotropic resolution for the volume TR, acceleration and other parameters we used. Reaching a thermal noise-dominated regime is usually desirable but enforces low SNR_0_, for which denoising strategies have been proposed (Vizioli et al., 2021).

Despite the physiological noise regime at the 1.2 mm resolution, we observe a trend of increased sensitivity to the DMN at 11.7T, detectable at individual level, compared to 7T at equivalent scan duration (Supplementary Fig. S5) and using the same nuisance regressors. Because our number of participants remains small and because we scanned different volunteers at 7T and 11.7T (scanners at different sites), our data are not sufficient to quantify with confidence the gain brought by the higher field strength. In both cases, parameters could be further optimized and future work include a thorough interfield comparison with further exploration of these parameters. For instance, we chose TE = 22 ms at 7T according to the HCP protocol which yet sought tradeoffs at 1.6 mm resolutions, while our TE = 19 ms at 11.7T was set according to literature values of $T_{2}^{*}$ and sequence timing constraints (which could be lifted using an improved gradient system; Feinberg et al., 2023) at the acceleration factor we used. As a result, the 7T data presented here was used as a quality control measure for the protocol and the analysis pipeline with a more ubiquitously found (commercial) instrument, while we make no claim of the optimality of the protocols at the two different field strengths. Moreover, we observe that the number of nuisance regressors can affect the comparison already at the same field strength. Using the 15 anatomical CompCor and 25 motion regressors seemed to return better DMN coverage without servoNav and PEERS while restricting them to the 25 motion parameters reversed the trend (Table 1). Given the evidence and literature suggesting that data variance in the physiological regime can contain network structure removable with nuisance regression (Bright & Murphy, 2015; Bright et al., 2017), our resting state results furthermore can hardly allow concluding on the benefits of *Servo on + PEERS* in connectivity analysis, despite evident tSNR gains. This naturally raises the question about the optimal denoising pipeline in resting state data analysis, especially when PMC schemes are employed. While servo navigation and PEERS’ corrections are probably too coarse to capture spatially-dependent neural activations, further investigation could focus on their impact on nuisance regressors, which are calculated on brain-wide masks. Task-based fMRI metrics (which are less susceptible to physiological noise), on the other hand, provide a more convincing demonstration of the gains brought by servoNav and PEERS, notably showing larger tSNR (Fig. 4), cleaner %ΔBOLD time courses (Fig. 8) and more significant activations for calculation, visual (Supplementary Fig. S6) and reading tasks, while motor task did not reveal consistently more intense activations. In general, the task-based results returned activated voxels in the expected regions and consistently across volunteers. Future work should aim to increase the resolution in order to take advantage of the thermal noise-dominated regime and potentially explore cortical layers (L. Huber et al., 2017) at 11.7T.

## 5 Conclusion

We report the first human resting-state and task-based fMRI study at 11.7T employing servo navigation and phase equalization to evaluate their gain. A preliminary comparison with 7T data using the same sequence and protocol (except for TE) is provided for resting-state and tSNR quality controls. Concrete metrics such as tSNR and z-scores in task-based experiments provide strong evidence that servoNav and PEERS benefit fMRI experiments, despite no clear gain in the detection of the DMN in resting-state and the optimal analysis pipeline question remaining. These first measurements were performed at moderately high 1.2 mm isotropic resolution as a first test of human fMRI on Iseult and further work will explore finer spatial (and/or temporal) resolution. This was considered an important milestone to verify the feasibility, stability and reliability of the Iseult 11.7T MRI scanner for fMRI given all the challenges we faced. The work furthermore highlights the importance of reducing vibration-induced eddy currents which increase with field strength and which can lead to disastrous tSNR losses. This work paves the way for human fMRI neuroscience studies at 11.7T and higher spatio-temporal resolution.

## Supplementary Material

Supplementary Material

## Data Availability

Unprocessed 11.7T MRI data available at https://doi.org/10.5281/zenodo.21993131. Preprocessing and analysis scripts available at https://github.com/Zaineb18/tristan_pipeline.
