# Supplementary material for: Human fMRI at 11.7T: Assessing feasibility, stability, and reliability on the Iseult scanner
Source: Imaging Neurosci (Camb). 2026 Sep 17;4:IMAG.a.1362. doi: 10.1162/IMAG.a.1362 (PMC13588317; doi:10.1162/IMAG.a.1362)
Supplement: Supplementary file 1 [file IMAG.a.1362_supp.pdf]

Human fMRI at 11.7T: Assessing Feasibility, Stability, and Reliability on the Iseult Scanner

Joseph Obriot[1†], Zaineb Amor[2,3†], Matthias Serger[4],
Rüdiger Stirnberg[4], Philipp Ehses[4], Malte Riedel[5], Tony Stöcker[4],
Klass P Prüssmann[5], Shajan Gunamony[6], Son Chu[6], Alexis Amadon[1],
Alexandre Vignaud[1], Vincent Gras[1], Florent Meyniel[2,3],
Franck Mauconduit[1], Caroline Le Ster[1], Nicolas Boulant[1*]

[1]Université Paris-Saclay, CEA, CNRS, BAOBAB, Neurospin, Gif-sur-Yvette, France

[2]Institut de neuromodulation, GHU Paris, psychiatrie et neurosciences, centre hospitalier Sainte-Anne, pôle hospitalo-universitaire 15, Université Paris Cité, Paris, France

[3]Cognitive Neuroimaging Unit, INSERM, CEA, Université Paris-Saclay, NeuroSpin center, Gif-sur-Yvette, France

[4]German Center for Neurodegenerative Diseases (DZNE), Bonn, Germany

[5]Institute for Biomedical Engineering, ETH Zurich and University of Zurich, Zurich, Switzerland

[6]Imaging Center for Excellence, University of Glasgow, Glasgow, United Kingdom

*Correspondence: nicolas.BOULANT@cea.fr

†Equal contribution: both authors may report this publication as first author on their CVs.

July 31, 2026

Supplementary Material

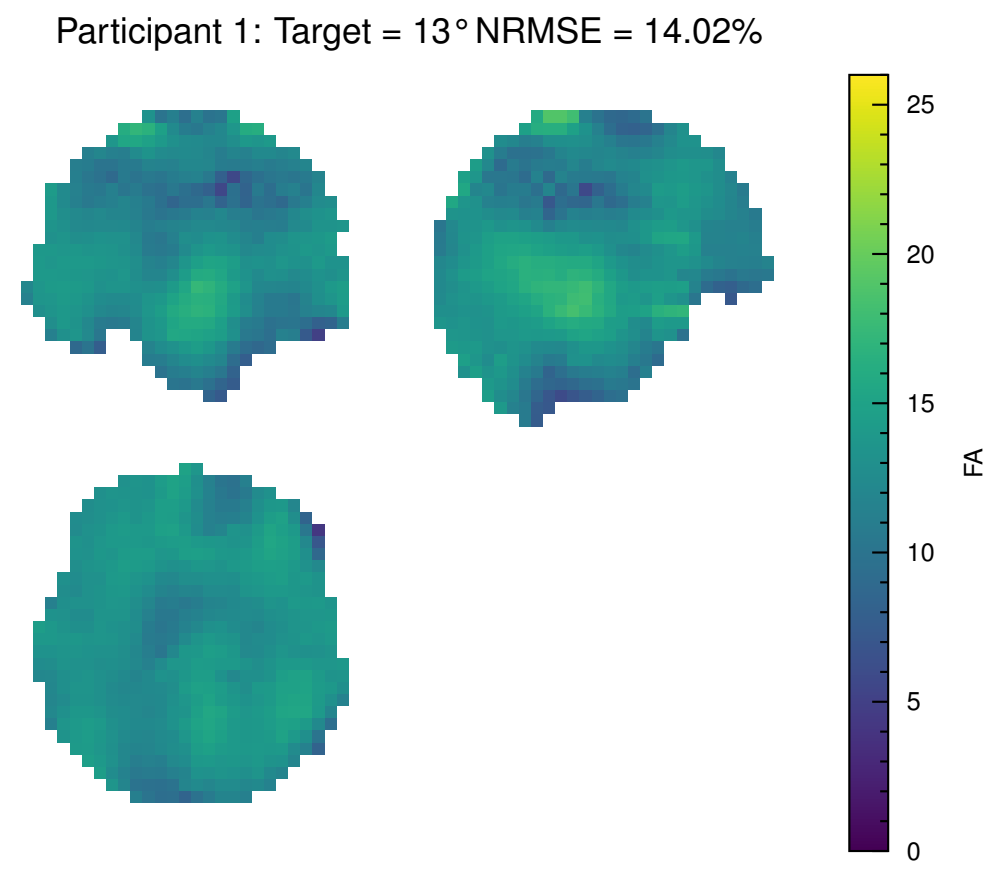

Figure S1: **Flip angle map of a representative participant.**
FA map of participant 1. FA-NRMSE was 14.02%, which is close to the 11.7T group mean FA-NRMSE of this study.

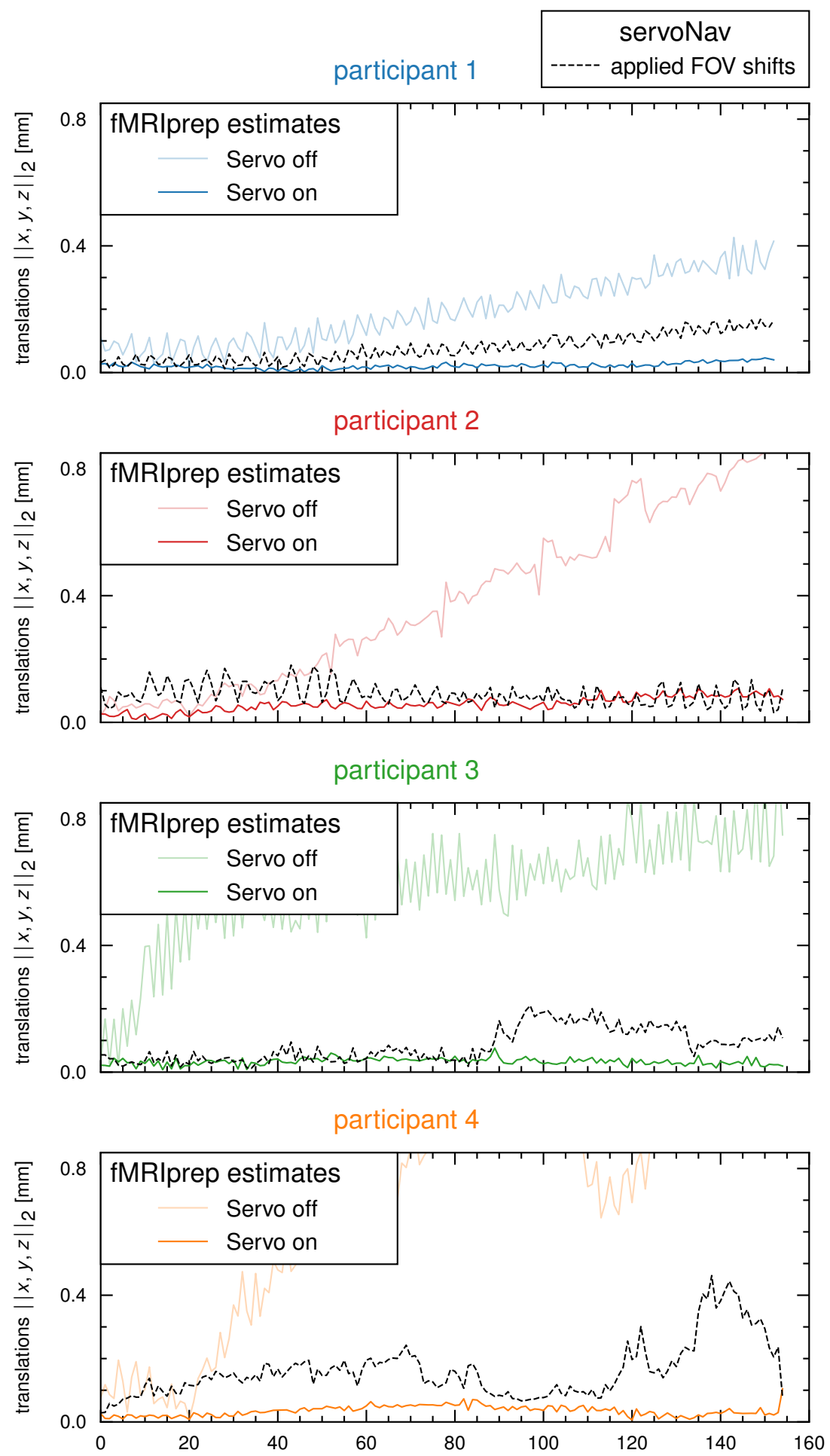

	RMS displacement [mm]
Servo off	0.22
Servo on	0.02

	RMS displacement [mm]
Servo off	0.47
Servo on	0.06

	RMS displacement [mm]
Servo off	0.61
Servo on	0.04

	RMS displacement [mm]
Servo off	0.79
Servo on	0.04

Group:

	RMS displacement [mm]
Servo off	0.52
Servo on	0.04

Figure S2: **Motion traces of the 11.7T task data.**
Translation estimates of the participants in localizer runs, rotations have not been plotted to avoid cluttering the figure. The residual motion computed during preprocessing is shown in color for the servo-navigated runs (vivid) and without PMC runs (faded). The FOV shifts applied in PMC are shown in dashed black lines.

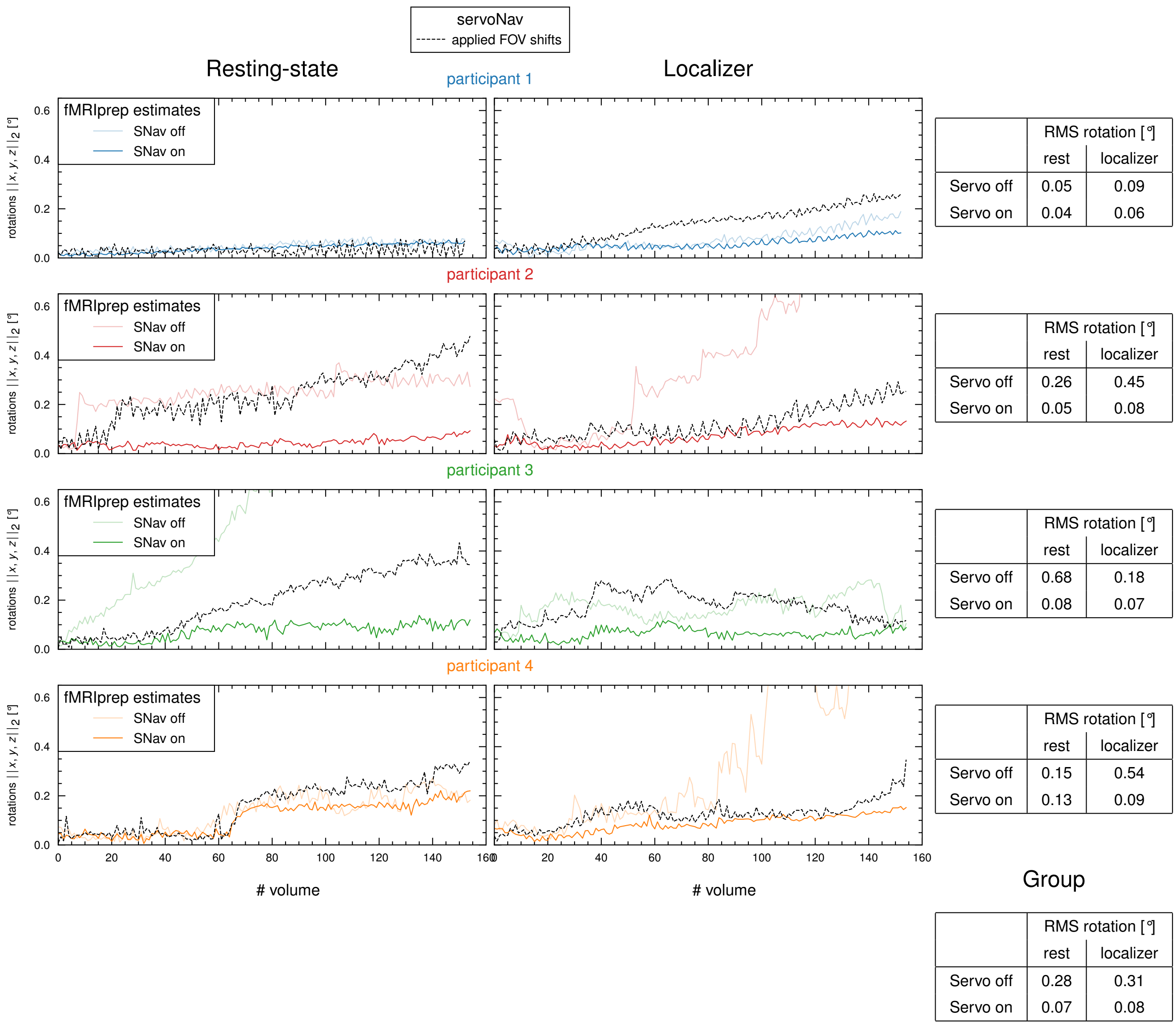

Figure S3: **Rotation traces of the 11.7T resting-state and task data.**
Rotation estimates of the participants in resting-state (left) and localizer (right) runs. The residual motion computed during preprocessing is shown in color for the servo-navigated runs (vivid) and without PMC runs (faded). The FOV shifts applied in PMC are shown in dashed black lines.

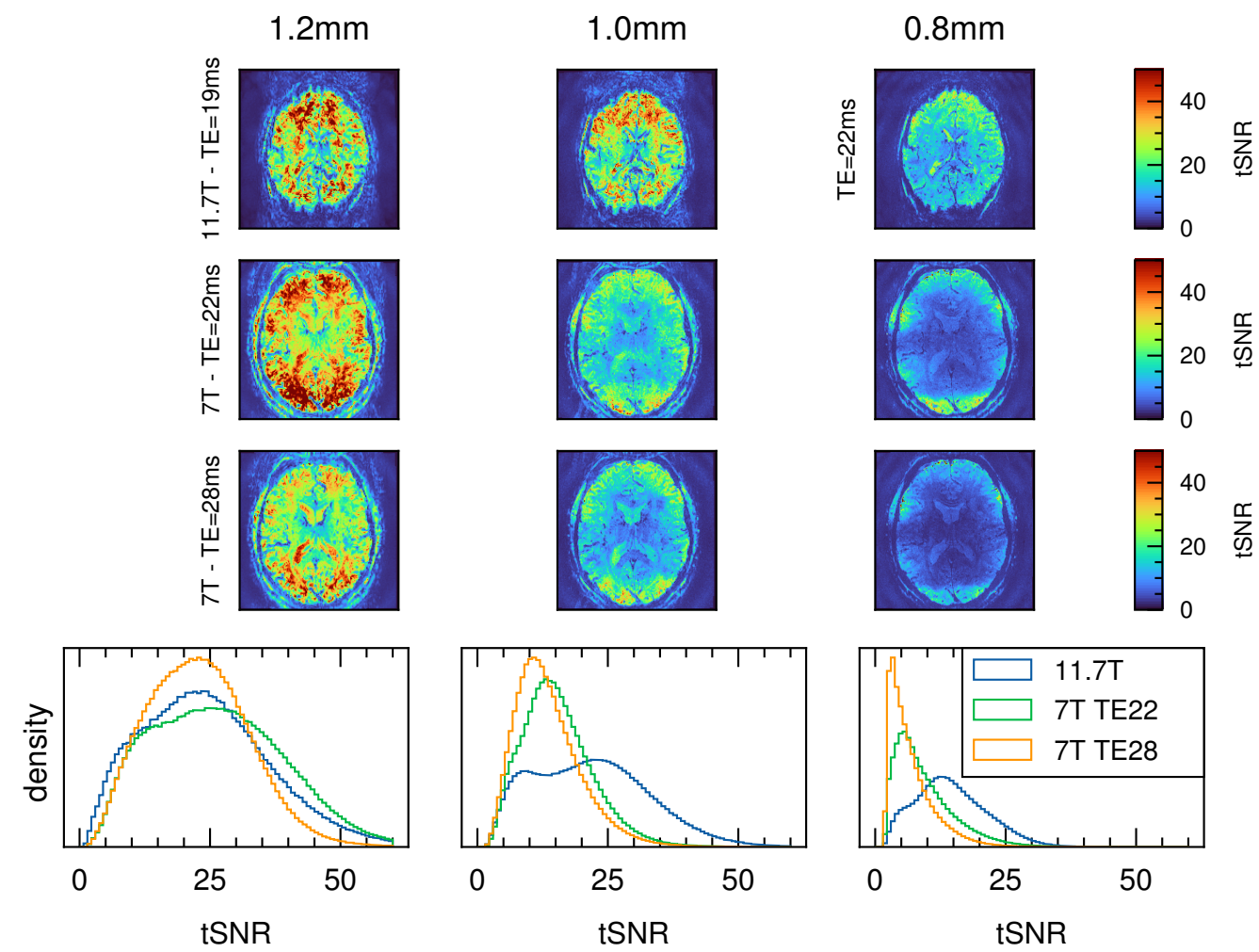

Figure S4: **Temporal SNR at 11.7T and 7T and increasing resolution.**
A single participant per field strength (different from **Figure ?? B**) scanned at increasing resolution revealing the crown-like pattern of tSNR – already present at 7T and 1.2 mm – appearing at a higher resolution at 11.7T (around 1 mm).

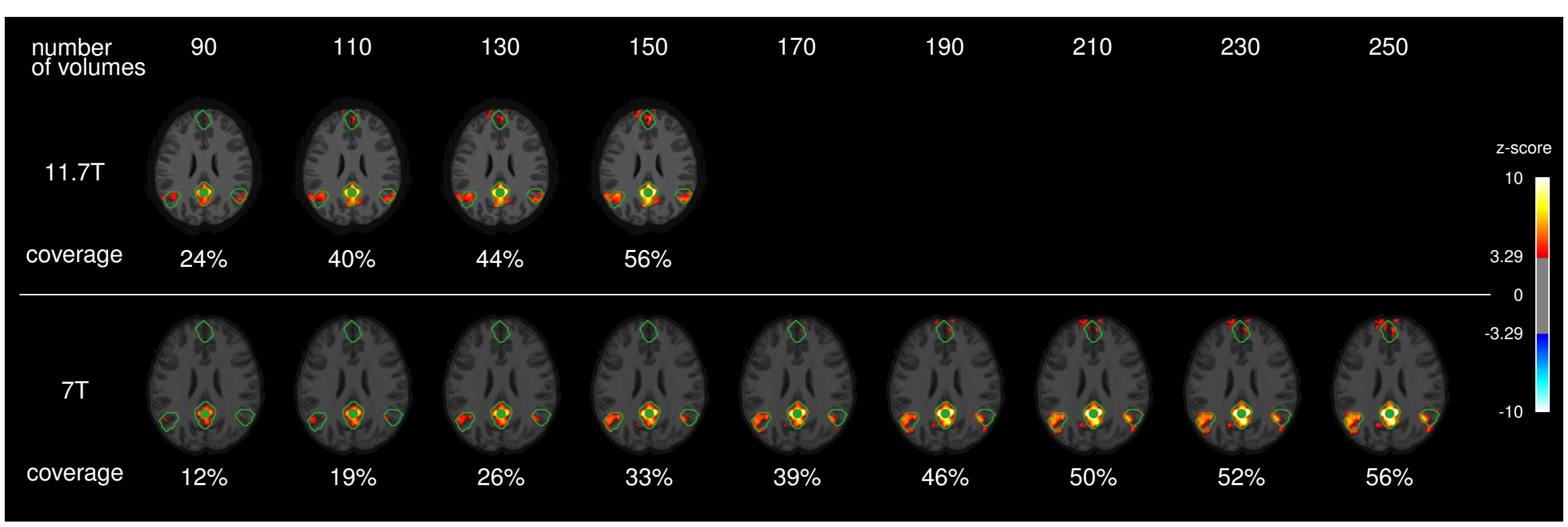

Figure S5: **Group DMN 11.7T vs 7T.**
Group maps and coverage obtained using increasing number of volumes in the resting-state analysis. The DMN nodes used to calculate coverage are contoured in green. The seed in the PCC is indicated by a green dot.

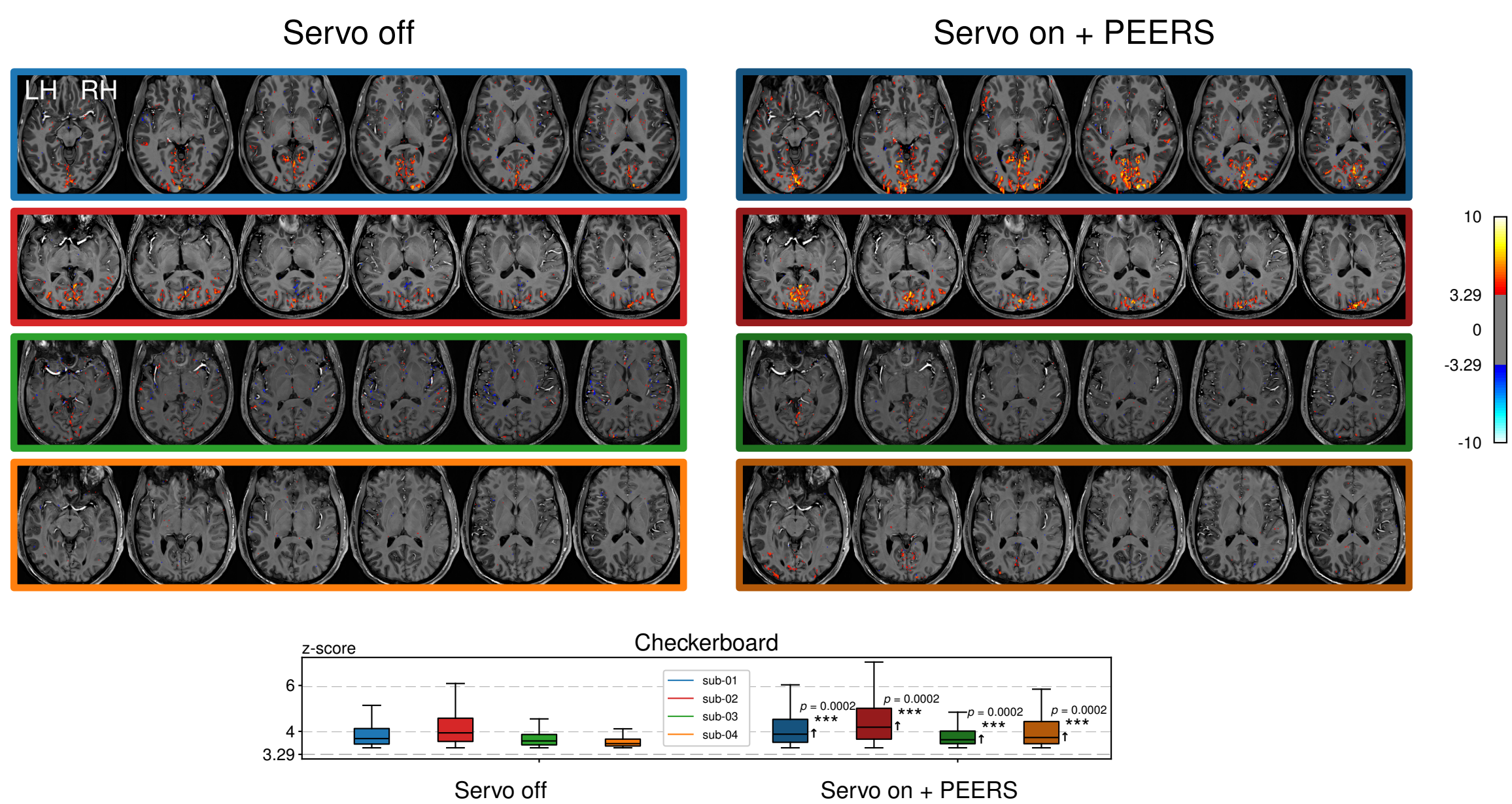

Figure S6: **Participant-wise visual activation maps at 11.7T in the T_1-w native space and boxplots of the associated significant z-scores distributions.**
Statistically significant (FPR at $p < 0.001$) z-score maps associated with the "Checkerboard" contrast. The slices shown represent the same slices in MNI space that were then projected back into each participant's native space in order to facilitate the between participant comparison in the native T_1-w space. From top to bottom: Participants 1–4. The median of statistically significant z-score (FPR at $p < 0.001$) values obtained from *Servo on + PEERS* data were compared to those obtained from the *Servo off* baseline data using permutation tests.

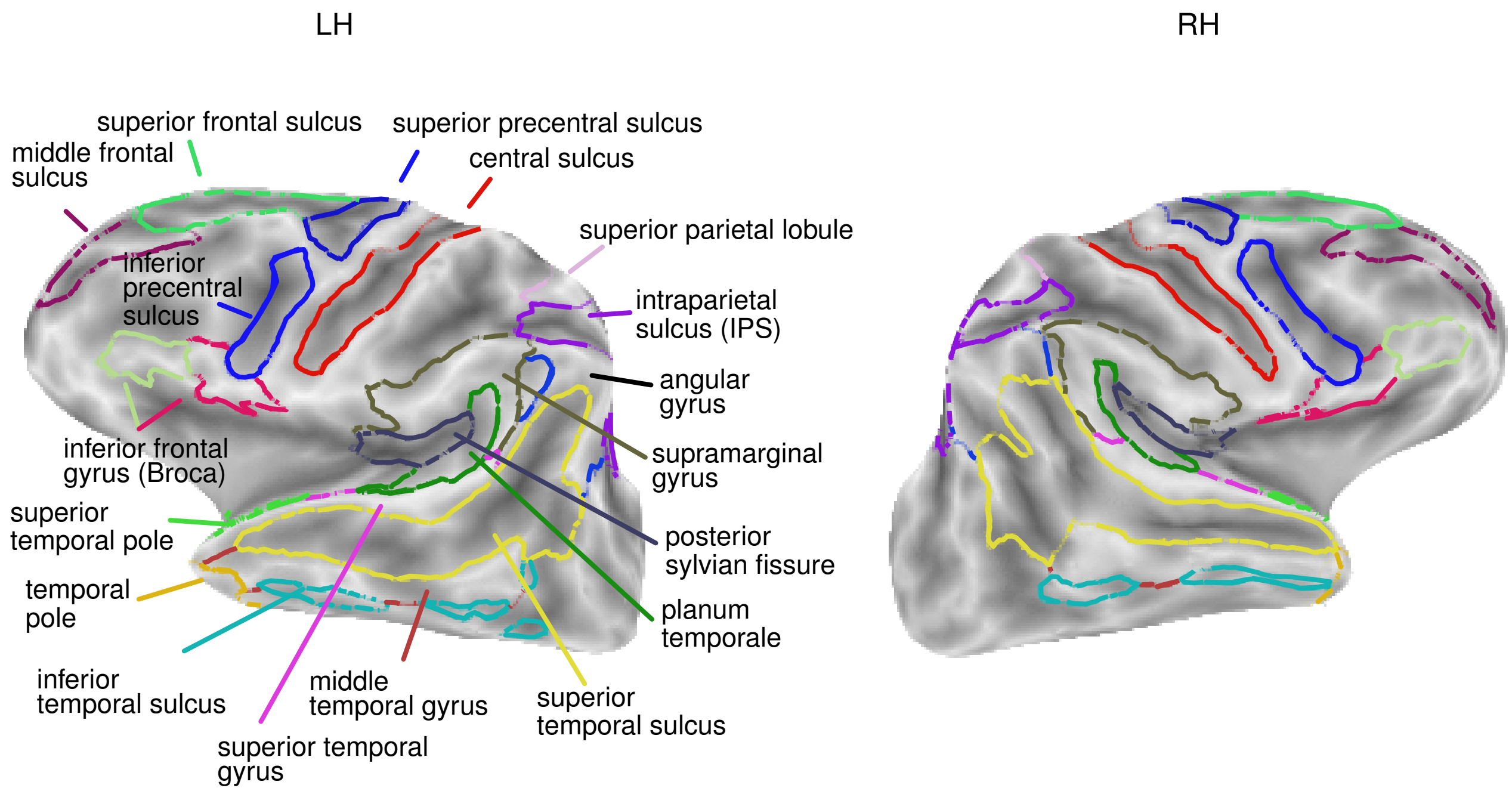

Figure S7: **Key for interpreting the Destrieux parcellations.**
Selected language and math-related parcels from the Destrieux atlas projected and overlaid onto the T1-weighted native cortical surface of participant 2.

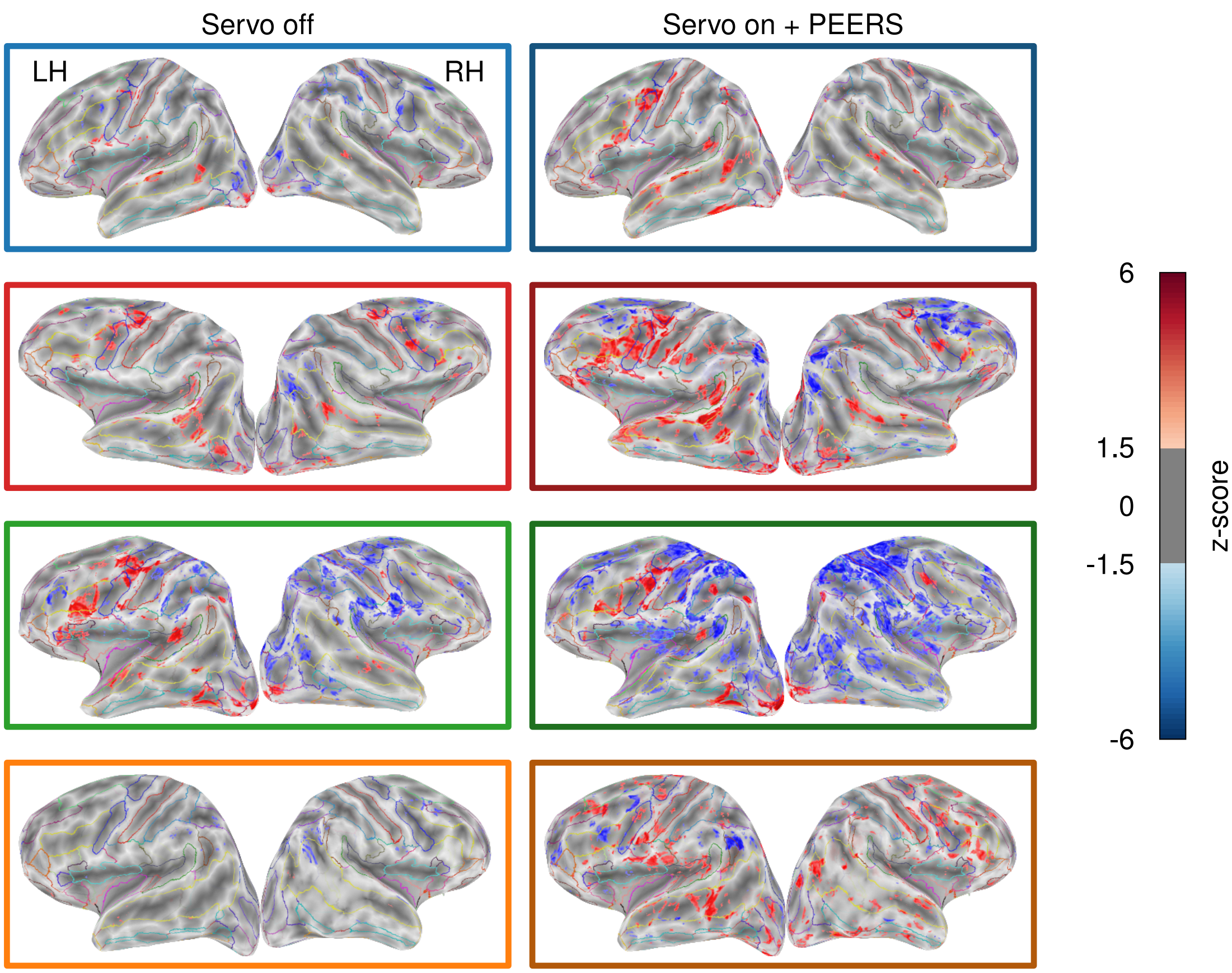

Figure S8: **Participant-wise language-related activation maps at 11.7T projected onto the native cortical surface.**
Participant-wise z-score maps for the ‘Phrases’ contrast, projected onto the cortical surface and thresholded using a less stringent threshold ($z > 1.5$). The contours of the parcels in Destrieux atlas projected into each participant’s native space are overlaid to the surface-projected activation patterns. From top to bottom: Participants 1–4.
